# Crosstalk between m^6^A modification and metabolic reprogramming in colorectal cancer: from the immune microenvironment to clinical applications

**DOI:** 10.3389/fimmu.2026.1911067

**Published:** 2026-08-12

**Authors:** Liang Zhao, Zhitao Yin, Chenchun Ji, Yan Wu, Hongjian Gao, Hexue Yuan

**Affiliations:** 1 Department of Colorectal Anal surgery, Shenyang Coloproctology Hospital, Shenyang, Liaoning, China; 2 Department of Gastrointestinal Oncology Surgery, Shenyang Coloproctology Hospital, Shenyang, Liaoning, China

**Keywords:** CRC, gut microbiota, m^6^A, metabolic reprogramming, TME

## Abstract

N6-methyladenosine (m^6^A) modification is the most common epigenetic alteration in eukaryotic mRNA, significantly impacting metabolic reprogramming and the tumor microenvironment (TME) of colorectal cancer (CRC) by dynamically regulating RNA metabolic processes. Recent studies indicate that m^6^A modification interacts with CRC metabolic reprogramming, fostering an immunosuppressive TME. Specifically, m^6^A modification promotes CRC progression by regulating glucose, lipid, and amino acid metabolism while inhibiting the activity of antitumor immune cells (such as T cells, natural killer cells, and macrophages) and activating tumor immunosuppressive cells (including tumor-associated macrophages, myeloid-derived suppressor cells, regulatory T cells, tumor-associated neutrophils, and cancer-associated fibroblasts). This article systematically reviews the molecular mechanisms through which m^6^A modification drives the malignant progression of CRC via metabolic regulation, elucidates the metabolic network involving m^6^A modification and its role in shaping TME, and discusses the clinical potential of targeting m^6^A modification and/or metabolic pathways, offering novel research avenues for CRC treatment.

## 1 Introduction

Colorectal cancer (CRC) is the third most prevalent malignant tumor globally, with a 5-year survival rate of < 15% for metastatic cases (1–3). Despite significant advancements in diagnostic and therapeutic techniques in recent years, the heterogeneity and metabolic plasticity of CRC continue to pose therapeutic challenges. Traditionally, oncogene activation and tumor suppressor gene inactivation have been considered the primary drivers of CRC development. However, recent research has highlighted metabolic reprogramming and immune evasion as crucial hallmarks of tumors (4), with further studies demonstrating that metabolic reprogramming not only promotes tumor progression but also contributes to the formation of an immunosuppressive tumor microenvironment (TME) (5).

**Figure 1:**
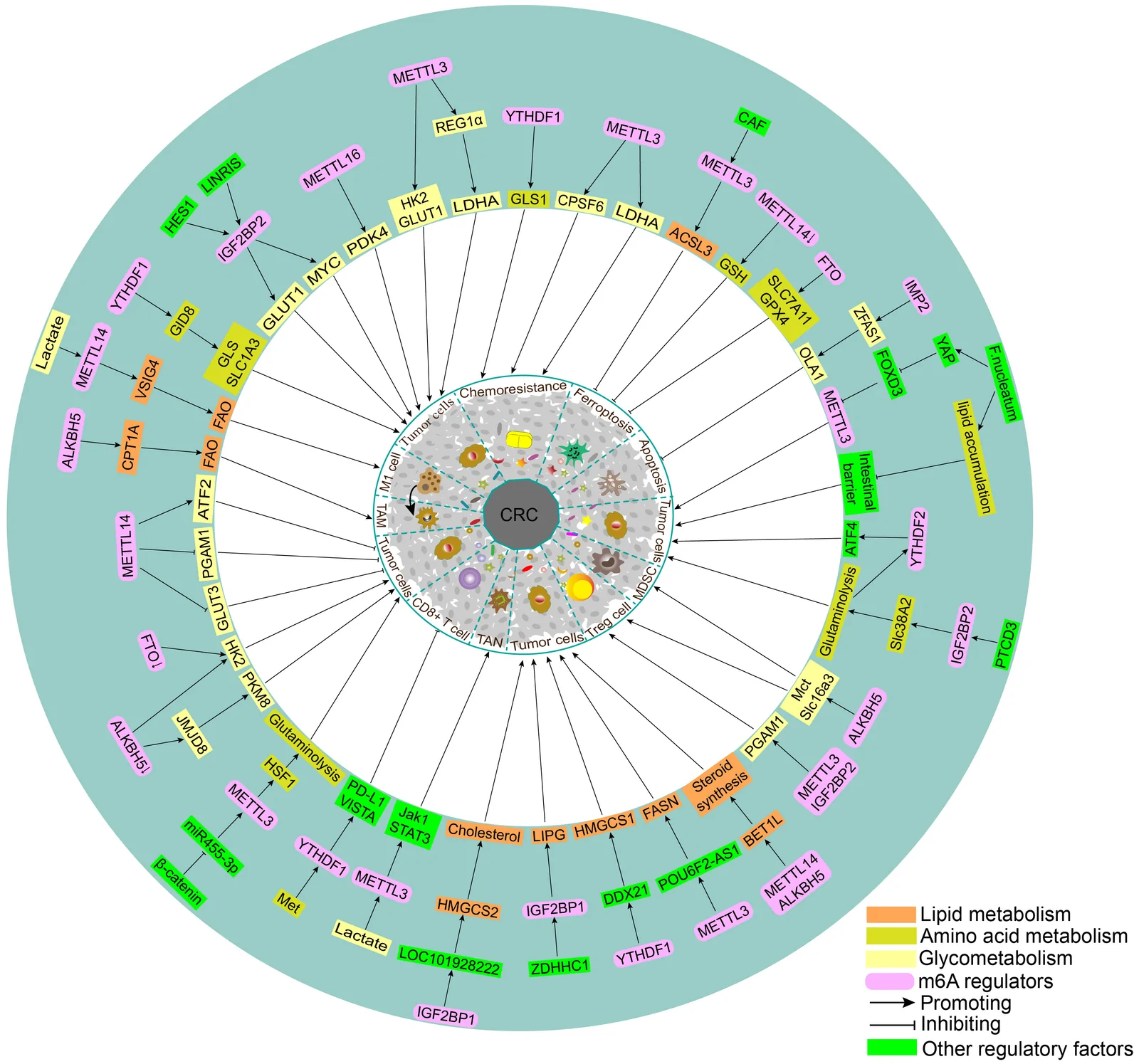
Effects of m^6^A modification on immune and tumor cells within the TME in CRC.

Metabolic alterations in CRC are characterized by enhanced glycolysis, increased glucose uptake and consumption, increased lipid and protein synthesis, and elevated amino acid uptake and catabolism. These metabolic shifts influence various aspects of cancer biology, including signaling pathways, tumor cell proliferation, invasion, metastasis, drug resistance, and the establishment of an immunosuppressive TME. Epigenetic modifications promote the progression of CRC (6, 7), especially, N6-methyladenosine (m^6^A), a methylation process occurring at the N6 position of adenosine, is a prominent and conserved internal transcriptional modification found in eukaryotic messenger RNAs, affecting RNA transcription, processing, splicing, degradation, and translation (8). This methylation is installed by methyltransferases, known as “writers” (such as methyltransferase-like (METTL) 3/14, Wilms tumor 1-associated protein (WTAP), RNA-binding motif protein 15/15B, Vir-like m6 A methyltransferase associated, and zinc finger CCCH-type containing 13), and removed by demethylating enzymes, referred to as “erasers” (including fat mass and obesity-associated protein (FTO), AlkB homolog (ALKBH 5, and ALKBH3). The m^6^A marks are recognized by m^6^A-binding proteins, termed “readers” (such as YTH domain-containing protein (YTHDC) 1/2, YTH N6-methyladenosine RNA-binding protein (YTHDF) 1/2/3, insulin-like growth factor-2 mRNA-binding protein (IGF2BP) 1/2/3, heterogeneous nuclear ribonucleoproteins (HNRNP), eukaryotic initiation factor 3 (eIF3), and insulin-like growth factor 2 mRNA-binding protein (IMP2) (8). Growing evidence indicates that m^6^A methylation significantly impacts tumor cell metabolism and plays a role in the pathogenesis of various diseases, particularly cancer (9, 10). Moreover, researchers have discovered that m^6^A modifications can regulate glucose (11, 12), lipid (13), and amino acid metabolism (14) in CRC, contributing to tumor progression and the formation of an immunosuppressive TME driven by metabolites. This review summarizes the mechanisms by which m^6^A promotes tumor progression through metabolic reprogramming in CRC and how m^6^A modification fosters the creation of an immunosuppressive TME via metabolites. We also explored the potential for future clinical applications targeting m^6^A and/or metabolic reprogramming in CRC (**Figure 1**).

## 2 M^6^A methylation promotes CRC progression through metabolic reprogramming

CRC cells adapt their metabolism to support rapid growth and metastasis. Large-scale studies suggest that m^6^A modification broadly influences cancer metabolic networks by directly regulating the expression of metabolic genes or metabolism-related signaling pathways, thus facilitating tumor progression (10, 15). We further examined how m^6^A modification advances CRC progression by modulating glucose, lipid, and amino acid metabolism in CRC.

### 2.1 M^6^A modification reprograms glucose metabolism to promote CRC progression

A defining characteristic of CRC is the increased uptake of glucose and production of lactate, even in aerobic conditions, a phenomenon referred to as the “Warburg effect” (16). Glycolysis plays a crucial role in CRC initiation and progression, influencing self-renewal, proliferation, and metastasis (17). Recently, m^6^A modification has garnered considerable attention in CRC research. METTL3 promotes CRC progression by regulating lactate dehydrogenase A (LDHA) expression through diverse pathways. For example, METTL3 modulates the expression of regenerative islet-derived protein 1α (REG1α) in an m^6^A-dependent manner mediated by MYC, which enhances LDHA activity and promotes the proliferation, migration, and invasion of CRC cells (18). Similarly, METTL3 regulates the expression of cleavage and polyadenylation specificity factor 6 and hypoxia-inducible factor 1α (HIF-1α) in an m^6^A-dependent manner, thereby increasing LDHA activity and stimulating glucose uptake and lactate production in cancer cells, ultimately promoting CRC growth (19, 20). Furthermore, METTL3-m^6^A modification regulates glucose transporter 1 (GLUT1) and hexokinase 2 (HK2) expression, activating glycolysis and promoting CRC progression (21, 22). Additionally, METTL16-mediated m^6^A modification enhances the mRNA stability of “suppressor of glucose by autophagy,” which upregulates pyruvate dehydrogenase kinase 4 (PDK4) and activates glycolysis to promote CRC progression (11). Meanwhile, METTL14-mediated m^6^A modification positively regulates activator of transcription factor (ATF) 2 (23) and negatively regulates solute carrier family 2 member 3 (GLUT3) and phosphoglycerate mutase (PGAM) expression (24), thereby inhibiting glycolysis and suppressing CRC cell proliferation. Collectively, these studies illustrate the diversity and complexity of m^6^A modification and methyltransferase expression in CRC.

In CRC occurrence and progression, insulin-like growth factor 2 mRNA-binding protein 2 (IGF2BP2) and IMP function as oncogenic m^6^A readers. For instance, IGF2BP2 enhances the stability of m^6^A-modified GLUT1 mRNA (25), increasing glycolysis, and promotes aerobic glycolysis in CRC via MYC-mediated pathways, facilitating CRC progression (26). In addition, IMP2 stabilizes the expression of the long non-coding RNA (lncRNA) ZNFX1 antisense RNA 1 in an m^6^A-dependent manner, thereby enhancing obg-like ATPase 1 activity and promoting glycolysis (27). This leads to increased CRC cell proliferation, colony formation, and apoptosis inhibition (27). In contrast, FTO and ALKBH5 negatively regulate glycolytic activity in CRC, thereby suppressing tumor progression. Specifically, the downregulation of FTO and ALKBH5 stabilizes m^6^A-modified HK2 mRNA expression (28) and upregulates pyruvate kinase M2 (PKM2) enzymatic activity by increasing Jumonji domain-containing protein 8 expression (29), thus promoting CRC glycolysis and tumor progression.

These studies underscore m^6^A modification as a crucial regulator of CRC initiation and progression, influencing glycolytic metabolism through diverse m^6^A-dependent mechanisms that either promote or suppress biological functions in CRC cells. A more comprehensive understanding of these mechanisms may lead to the identification of novel therapeutic targets and innovative approaches for CRC treatment.

### 2.2 M^6^A modification reprograms lipid metabolism to promote CRC progression

Another hallmark of CRC is the abnormal reprogramming of lipid metabolism, which serves as a critical driver of tumor progression (30, 31). Dysregulated lipid metabolism in cancer cells disrupts membrane integrity, energy balance, signaling pathways, and gene expression, ultimately affecting cellular functions and fostering malignant phenotypes (31). Emerging research highlights the pivotal role of m^6^A RNA methylation in reprogramming lipid metabolism in CRC, driving tumor progression through multiple mechanisms. In particular, the m^6^A reader protein IGF2BP1 collaborates with LOC101928222 to stabilize the mRNA of 3-hydroxy-3-methylglutaryl-CoA synthase 2 (HMGCS2)—a key enzyme in cholesterol synthesis—in an m^6^A-dependent manner, thereby enhancing cholesterol production and accelerating CRC development (32). Conversely, the tumor suppressor zinc finger DHHC-type containing 1 inhibits CRC growth by reducing lipase G expression via IGF2BP1, which also relies on m^6^A modification (13). Furthermore, the m^6^A reader YTHDF1 directly activates HMGCS1, another crucial enzyme in cholesterol synthesis, by stabilizing DEAD-box helicase (DDX) 21 expression, further regulating lipid metabolism to drive tumor progression (33). Notably, m^6^A modification influences not only the classical cholesterol pathway but also fatty acid metabolism by regulating non-coding RNAs and gene expression. For instance, METTL3-mediated m^6^A modification upregulates POU class 6 homeobox 2 antisense RNA 1, a lncRNA, prompting Y-box binding protein 1 to bind to the fatty acid synthase (FASN) promoter and enhance its transcription, thereby promoting fatty acid metabolism and growth in CRC cells (34). Furthermore, the single-nucleotide polymorphism rs11245997 reduces m^6^A levels in the 3’ untranslated region of the blocked early in transport 1 homolog (yeast)-like (BET1L) gene, disrupting miR-140-3p binding and consequently upregulating BET1L expression (35). This activation of the steroid synthesis pathway increases CRC risk (35).

Overall, m^6^A modification significantly reprograms lipid metabolism in CRC by precisely regulating key genes and RNA stability involved in cholesterol and fatty acid synthesis and oxidation, as well as related signaling pathways. This not only promotes tumorigenesis and progression but also reveals the immense potential of targeting m^6^A modification and lipid metabolism as vital therapeutic strategies against CRC.

### 2.3 M^6^A modification reprograms amino acid metabolism to promote CRC progression

In recent years, the pivotal role of m^6^A RNA methylation in regulating amino acid metabolism in CRC has become increasingly clear, shedding light on its malignant progression. Amino acid metabolic reprogramming, particularly abnormal glutamine catabolism, is a defining feature of CRC. Glutamine is converted to glutamate via glutaminase (GLS) catalysis and subsequently processed by glutamate dehydrogenase to produce α-ketoglutarate (α-KG), which enters the tricarboxylic acid cycle. This pathway supplies CRC cells with energy and biosynthetic precursors, contributing to what is termed “glutamine addiction,” which significantly enhances tumor proliferation and metastasis (36–38). Evidence suggests that m^6^A modification finely regulates this metabolic process through various mechanisms. For instance, lysine acetyltransferase 2A epigenetically upregulates pentatricopeptide repeat domain 3 expression, thus enhancing the stability of solute carrier family 38 member 2 mRNA—a glutamine transporter—via IGF2BP2 in an m^6^A-dependent manner, thereby increasing glutamine uptake and metabolism to promote CRC metastasis (39). Additionally, β-catenin inhibits miR-455-3p production, subsequently promoting heat shock factor 1 expression through METTL3-m^6^A-mediated mechanisms, accelerating glutamine catabolism and CRC progression (40). Furthermore, YTHDF1-mediated m^6^A recognition upregulates solute carrier family 1 member 3 and GLS expression by enhancing the translation of glucose-induced degradation protein 8 homolog, sustaining glutamine metabolic activity and driving CRC malignancy (41). Consequently, inhibition of glutamine catabolism has emerged as a potential therapeutic target in CRC. However, challenges exist in this approach. Inhibiting glutaminolysis can lead to an upregulation of ATF4 expression via YTHDF2-dependent m^6^A recognition, activating DNA damage-inducible transcript 4 and inducing pro-survival autophagy (42), ultimately diminishing the antitumor efficacy of glutaminolysis inhibition (42). Furthermore, m^6^A modification also influences CRC progression by regulating ferroptosis. For example, FTO and METTL14 independently regulate the expression of solute carrier family 7 member 11 (SLC7A11) or glutathione peroxidase 4 (GPX4) via YTHDF2-dependent mechanisms, promoting glutathione synthesis and lipid peroxide clearance to protect CRC cells from ferroptosis (43, 44). In terms of drug resistance, YTHDF1-mediated m^6^A modification enhances GLS1 protein synthesis, which contributes to CRC cell resistance to cisplatin and oxaliplatin (14, 45).

These findings elucidate the molecular mechanisms by which m^6^A modification drives CRC progression through multilayered regulation of amino acid metabolism, providing a theoretical foundation for developing novel therapeutic strategies targeting the m^6^A-mediated amino acid metabolism axis.

### 2.4 Crosstalk among gut microbiota, m^6^A modification, metabolites, and TME: promoting CRC progression

Microbiota–host interactions are essential in CRC progression, though the mechanisms involved remain unclear. Microbes/metabolites can induce driving mutations (such as DNA damage and signal disruption) and alter epigenetic factors (such as DNA methylation, histone modifications, and non-coding RNAs), thereby promoting the proliferation and survival of tumor cells (46). Research has established a strong correlation between gut microbiota and the production and utilization of methyl donors in the human body. Methyl groups, primarily generated through one-carbon metabolism pathways, participate in m^6^A RNA methylation via the folate and methionine cycles. These methyl group sources are termed methyl donors (47). They can be obtained through diet or produced by gut microbiota, with their metabolism influenced by the microbiome. For instance, non-starch polysaccharides can modulate m^6^A RNA methylation by altering the supply of methyl donors from the gut microbiota, potentially alleviating cancer (48). Furthermore, butyrate—a short-chain fatty acid (SCFA) produced by gut microbiota—reduces METTL3 expression and m^6^A levels in CRC cells, thus inhibiting tumor progression (49). Conversely, Chen S et al. (50) discovered that *Fusobacterium nucleatum* suppresses METTL3 by activating the Yes-associated protein (YAP) signaling pathway, which inhibits Forkhead box D3(FOXD3)expression and increases CRC invasiveness. These findings present seemingly contradictory views on METTL3 downregulation. However, studies indicate that Fusobacterium nucleatum can disrupt the intestinal barrier, thereby promoting tumor growth (51). Furthermore, FOXD3 also suppresses CRC progression by increasing miR-133a expression, which in turn reduces UBA2 expression (52). The above studies demonstrate the complexity and diversity of the Fusobacterium nucleatum/FOXD3/METTL3 pathway, which may further explain the discrepancies among the aforementioned research findings. Overall, the diverse and complex interactions between gut microbiota and the host influence CRC growth via multiple mechanisms.

The gut microbiota not only influences m^6^A modification but also modulates metabolic reprogramming, thereby promoting tumor progression. Metabolites constitute a crucial component of the interaction between the gut microbiome and the human body (53). Microorganisms in the colon produce metabolites after ingesting specific substances, which serve as signaling molecules and substrates in metabolic reactions (54) and are closely linked to CRC development (55). In patients with CRC, increased *F. nucleatum* levels are associated with lipid accumulation, which exacerbates inflammation and disrupts the intestinal barrier, further promoting CRC growth (51). Notably, oral Chinese herbal formulations elevate SCFA levels through gut microbiota–host interactions, enhancing M1 macrophage polarization and CD8^+^ T cell infiltration, ultimately reshaping the CRC immune microenvironment (56). Furthermore, the gut microbiota’s regulation of tryptophan metabolism influences not only CRC growth (57) but also TME (58, 59). These studies underscore the significant role CRC-associated dysbiosis may play in impairing host metabolic reprogramming.

**Figure 2:**
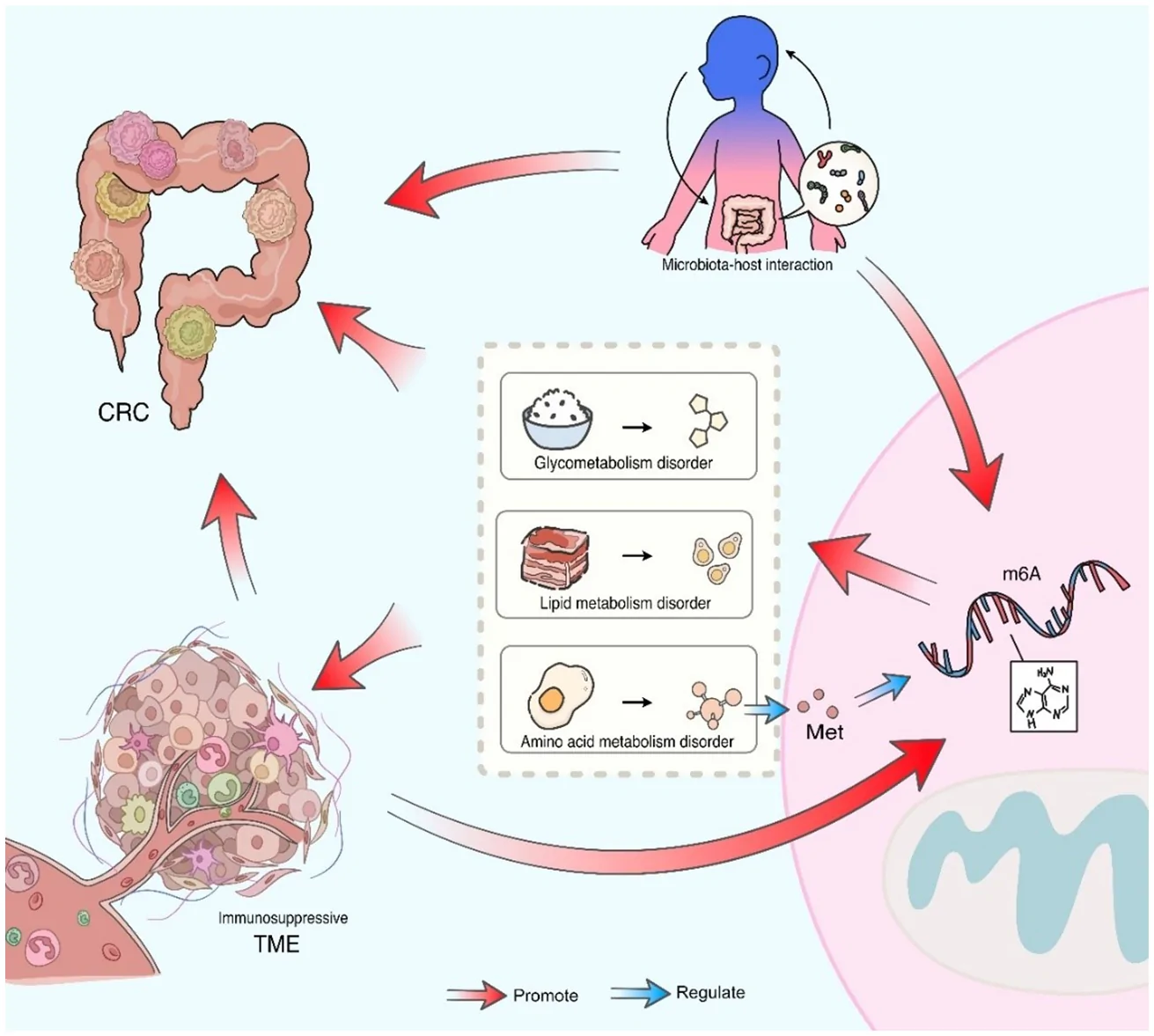
Crosstalk between the gut microbiota, m^6^A modification, metabolites and TME in CRC.

Therefore, considering the impact of gut microbiota–host interactions on m^6^A modification and metabolic reprogramming, we hypothesize that crosstalk among the gut microbiota, m^6^A modification, metabolites, and TME plays a critical role in CRC progression. Modulating the gut microbiota may offer a novel strategy for CRC prevention and treatment (**Figure 2**).

## 3 M^6^A modification reshapes the CRC immune microenvironment through metabolic reprogramming

As previously noted, m^6^A regulators modulate carbohydrate, amino acid, and lipid metabolism via m^6^A-dependent mechanisms, influencing biological functions in CRC cells. Concurrently, m^6^A modification extensively impacts TME across diverse cancers by regulating metabolic reprogramming, particularly in gastric cancer (60), bladder cancer (61), hepatocellular carcinoma (62), and CRC (63, 64). Therefore, we will focus on the pivotal role of the interplay among m^6^A modification, metabolism, and TME in establishing the immunosuppressive TME of CRC.

### 3.1 Interactions among m^6^A modification, metabolism, and antitumor immune cells in TME

#### 3.1.1 T cells

In TME, tumor and non-tumor cells exhibit increased lactate production owing to the Warburg effect, resulting in a highly acidic environment. This acidosis, similar to that observed in other cancers, impairs the activity of antitumor immune cells in CRC (65, 66). Notably, m^6^A modification regulates glucose metabolism in CRC and promotes tumor progression (19–22). Additionally, researchers have discovered that METTL3/IGF2BP2 modifies circular RNA QSOX1 (circQSOX1) via m^6^A, facilitating its interaction with miR-326/330-5p, which upregulates the expression levels of the glycolytic enzyme PGAM1 and the immune checkpoint molecule cytotoxic T lymphocyte-associated antigen-4 (CTLA-4), exacerbating immune evasion (67). Although literature on this is limited, we propose that m^6^A modification likely suppresses T cell activity by regulating glucose metabolism, thereby contributing to the development of an immunosuppressive TME in CRC.

Research indicates that CRC cells compete with CD8^+^ T cells for tryptophan, impairing CD8^+^ T cell function and promoting an immunosuppressive TME (68). Likewise, in CRC, the T-complex protein 1 subunit epsilon (chaperonin containing TCP1 subunit 5) enhances asparagine synthesis, thus reducing effector CD8^+^ T cell numbers and supporting CRC cell proliferation (69). Furthermore, CRC marked by elevated GLS expression exhibits diminished T cell infiltration and attenuated cytotoxicity, correlating with a poor prognosis (70). These findings underline the complex diversity of amino acid metabolism in CRC and its significant effects on T cells. Considering the indispensable role of m^6^A modification on CRC amino acid metabolism, we hypothesize that it likely regulates amino acid metabolism to influence T cell function. This hypothesis is reinforced by evidence that S-adenosylmethionine, a metabolite of methionine metabolism, promotes the translation of immune checkpoint molecules via YTHDF1 in an m^6^A-dependent manner, thereby suppressing CD8^+^ T cell infiltration and driving CRC progression (63). Collectively, these data suggest that m^6^A modification likely affects T cell function in CRC via metabolic reprogramming.

#### 3.1.2 Natural killer cells

NK cells, integral to the innate immune system, are vital for inhibiting tumor growth and preventing metastasis. In TME of CRC, a decline in NK cells and their impaired cytotoxic function significantly contribute to CRC progression (71). However, the exact regulatory mechanisms governing this phenomenon remain unclear. Recent studies have demonstrated that m^6^A modification influences NK cell migration and cytotoxicity. In CRC, for instance, METTL3 negatively regulates the secretion of C-X-C chemokine ligands 9 (CXCL9) and 10 (CXCL10) through an m^6^A-dependent mechanism, thereby inhibiting NK cell migration (72). Additionally, SMAD family member 4, a membrane receptor involved in SMAD signaling (73), enhances the expression of NK cell activation receptor “natural killer group 2, member D” by increasing YTHDF2-m^6^A levels, thus amplifying NK cell cytotoxicity against human CRC cells (71). Moreover, m6A modification reportedly regulates glucose metabolism to promote CRC progression. METTL3 increases the expression levels of HK2, solute carrier family 2 member 1 (GLUT1), and LDHA in CRC in an m^6^A-dependent manner, thereby boosting glycolysis and accelerating tumor development (22). Furthermore, studies have confirmed that glycolytic metabolism is upregulated in tumor cells from obese or overweight patients with CRC compared with that in their non-obese counterparts, impeding NK cell cytotoxic function and promoting tumor progression (74).

Consequently, m^6^A modification may plausibly influence NK cell function by regulating glucose metabolism. Given the complexities of metabolic interactions within CRC TME, m^6^A modification conceivably affects NK cell function by modulating other metabolic pathways. Notably, no comprehensive studies have yet addressed this aspect, necessitating further research to elucidate the intricate crosstalk among m^6^A modification, metabolism, and NK cells within CRC TME.

#### 3.1.3 Macrophages

Macrophages are multifunctional cells with unique functional characteristics shaped by their microenvironment in both *in vivo* and *in vitro* contexts. They can be categorized into two distinct types: M1 macrophages, which are classically activated macrophages that possess antitumor properties, and M2 macrophages, which are alternatively activated macrophages that exert pro-tumor effects. Extensive research indicates that m^6^A modification enhances glycolysis, culminating in increased lactate production in CRC (11, 19, 20). This glycolytic metabolite, lactate, significantly promotes M2 polarization of CRC macrophages, fostering an immunosuppressive TME (75). Moreover, lactate secreted by CRC cells upregulates the expression of V-set and immunoglobulin domain containing 4 (VSIG4) in macrophages via the histone H3 lysine 18 lactylation (H3K18la)–METTL14–m^6^A axis, which enhances fatty acid oxidation (FAO) in macrophages and drives M2 polarization, ultimately accelerating tumor progression (64). However, the researchers discovered that METTL14 inhibits the progression of CRC through glycometabolism (24), which is contrary to the aforementioned study. These findings indicate that m6A modification affects glucose metabolism in colorectal cancer through multiple mechanisms, or promotes the M2 polarization of macrophages, thereby facilitating tumor progression. Additionally, several studies have confirmed that CRC fatty acid metabolism also promotes M2 polarization in macrophages, thereby contributing to tumor progression. For example, a mouse model of fatty liver disease with induced CRC demonstrated that extracellular vesicles from fatty liver cells enhance macrophage fatty acid metabolism via mitogen-activated protein kinase-associated protein 1, leading to M2 macrophage polarization and aggravating CRC progression (76). Moreover, m^6^A modification significantly affects CRC fatty acid metabolism (13, 34), suggesting that it may promote M2 polarization of CRC macrophages via fatty acid metabolism, thereby driving tumor progression. In a co-culture model of CRC cells and macrophages, ALKBH5 was found to promote macrophage M2 polarization by demethylating m^6^A modifications, which upregulates carnitine palmitoyltransferase 1A (CPT1A) and enhances macrophage FAO, thus promoting CRC cell proliferation (77).

These insights imply that m^6^A modification may directly influence macrophage phenotypes in CRC by regulating glucose and lipid metabolism, creating an immunosuppressive TME. Consequently, targeting m^6^A regulators or key molecules involved in glucose and lipid metabolism could represent novel strategies for reversing the immunosuppressive microenvironment in CRC, offering potential avenues for combined metabolic intervention and immunotherapy.

### 3.2 Crosstalk among m^6^A modification, metabolism, and immunosuppressive cells in TME

#### 3.2.1 Tumor-associated macrophages

TAMs typically adopt an M2-like phenotype in response to their environment, establishing an immunosuppressive niche within TME (78). A recent study investigated the relationship between m^6^A modification and TAMs. Specifically, knockdown of METTL3 reduces miR-146b expression in an m^6^A-dependent manner, which enhances phosphoinositide 3-kinase (PI3K)/protein kinase B (AKT) signaling, promoting M2 polarization of TAMs and exacerbating immune suppression, ultimately driving CRC progression (79). Furthermore, a substantial body of literature indicates that m^6^A modification regulates glycolysis, leading to lactate accumulation and furthering CRC progression (11, 19, 20). Notably, CRC-induced lactate production increases signal-regulated protein alpha levels by activating the Ap-2α/Ets-like protein-1 (Elk-1) axis, thereby suppressing the antitumor activity of M1-polarized TAMs and enhancing the immunosuppressive activity of M2-polarized TAMs (80). These findings suggest that m^6^A modification promotes M2-TAM phenotypic conversion by regulating glucose metabolism, contributing to an inhibitory TME. Moreover, lactate secreted by CRC cells upregulates VSIG4 mRNA expression in macrophages through an m^6^A-dependent pathway mediated by METTL3 (64). This process stimulates FAO in macrophages, further driving their polarization toward the M2 phenotype of TAMs and advancing tumor progression (64). This finding corroborates the aforementioned perspective.

Currently, m^6^A modification is widely recognized to regulate lipid metabolism to promote CRC progression (32–34). Simultaneously, the high levels of growth differentiation factor 15 produced by macrophages enhance fatty acid β-oxidation, thereby promoting M2-TAM phenotypic conversion and decreasing CRC chemotherapy sensitivity (81). Furthermore, existing literature confirms that m^6^A modification promotes M2 polarization of TAMs in CRC by regulating lipid metabolism, thus advancing tumor progression. In co-culture models of CRC cells and macrophages, ALKBH5 was observed to regulate CPT1A expression in an m^6^A-dependent manner, thereby driving M2-TAM phenotypic transformation and stimulating CRC cell proliferation (77).

Although limited studies have demonstrated that m^6^A modification facilitates M2-TAM phenotypic conversion by regulating glucose and lipid metabolism, these findings highlight the central role of m^6^A modification in metabolic–immune cross-regulation and provide novel insights for formulating TAM-targeted therapeutic strategies.

#### 3.2.2 Myeloid-derived suppressor cells

MDSCs arise from bone marrow progenitor and immature myeloid cells, constituting a major component of TME and playing a vital role in regulating solid tumor progression and metastasis. MDSCs not only facilitate tumor cell proliferation, survival, and migration within TME but also suppress the immune responses of T, NK, and B cells (82). *Candida tropicalis* reportedly promotes colorectal tumorigenesis by activating the Syk–PKM2–HIF-1α glycolytic signaling axis in CRC, which enhances the immunosuppressive functions of MDSCs and increases their accumulation (83). Mechanistically, lactate—a key glycolytic byproduct—serves an essential role in MDSC infiltration (84). It not only recruits MDSCs (85) but also induces their formation (84) and maturation (86), ultimately suppressing antitumor immune responses. These findings suggest a link between elevated lactate levels and MDSCs in CRC. Additionally, m^6^A modification evidently facilitates MDSC recruitment (87). In CRC, METTL3 enhances MDSC migration by increasing the expression of basic helix–loop–helix family member E41 in an m^6^A-dependent manner, which induces CXCL1 transcription (87). Several studies have also explored the interplay among m^6^A modification, glucose metabolism, and MDSCs in CRC. For instance, ALKBH5 promotes glycolysis and lactate production by regulating the expression of monocarboxylate transporter (MCT) 4/solute carrier family 16 member 3 (SLC16A3), leading to increased MDSC levels and immunosuppressive TME formation (12). Furthermore, lactate in TME can induce the upregulation of METTL3 in MDSCs through H3K18 lactylation, enhancing Janus kinase 1 (JAK1) protein translation and subsequent signal transducer and activator of transcription 3 (STAT3) phosphorylation, thereby further promoting MDSC-driven immunosuppression (88). These studies illustrate the intricate relationship between m^6^A modification and MDSCs via glycolysis, offering insights for potential immunotherapy strategies in CRC.

#### 3.2.3 Regulatory T cells

Tregs are essential for maintaining immune homeostasis and establishing immune tolerance, particularly in regulating tumor immunity. In TME, Tregs are induced and differentiated by conventional T cells, exhibiting potent immunosuppressive activity that promotes tumor initiation and progression (89). They also suppress immune effector cell function through multiple mechanisms, rendering them pivotal in tumor immune escape (90, 91). Lactate, a byproduct of glycolysis, directly affects Treg recruitment to tumor sites (86, 92). It not only promotes induced-Treg development (92) and Treg proliferation (93, 94) but also increases the expression of programmed cell death protein 1 (PD-1) on Tregs in the hyperglycemic TME (95). Extensive research has established that m^6^A modification regulates glycolytic metabolism, thereby promoting CRC progression (11, 19, 20). Notably, two studies have linked m6A modification, glucose metabolism, and Tregs. First, ALKBH5 upregulates the expression of the lactate transporter MCT4/SLC16A3 in an m^6^A-dependent manner, facilitating lactate efflux and amplifying the immunosuppressive effects of Tregs, thereby promoting CRC cell growth (12). Second, under METTL3/IGF2BP2 mediation, m^6^A-modified circQSOX1 increases PGAM1 expression by sponging miR-326 and miR-330-5p, activating glycolysis (67). This process elevates CTLA-4 expression on Tregs, contributing to the formation of an immunosuppressive TME in CRC (67). These findings accentuate the crucial interplay among m^6^A modification, glycolysis, and Tregs in shaping the immunosuppressive TME, offering novel targets for tumor immunotherapy.

#### 3.2.4 Tumor-associated neutrophils

TANs constitute a vital component of the immunosuppressive TME and are known to promote tumor progression through various mechanisms, often correlating with poor prognosis (96). Despite their importance, discussions regarding the relationship between m^6^A modification and metabolism in TANs remain limited. Current studies have identified connections between m^6^A modification and TANs in lung (97) and thyroid (98) cancers. In CRC, research on the link between m^6^A modification and TANs is scarce, although associations have been observed between m^6^A modification and neutrophils in inflammatory bowel diseases. ALKBH5-deficient neutrophils exhibit decreased C-X-C motif chemokine receptor 2 expression, impairing their migration in response to the chemokine CXCL2 and diminishing overall neutrophil motility (99). Accordingly, we hypothesize that m^6^A modification potentially correlates with TANs in CRC. Furthermore, METTL3 increases LDHA expression in an m^6^A-dependent manner, leading to glycolytic reprogramming in CRC (20). This LDHA overexpression enhances glycolytic activity, inducing immunosuppressive and pro-tumor functions in neutrophil-like differentiated HL-60 cells (100). Although the association among m^6^A modification, glucose metabolism, and TANs in CRC TME appears interconnected, one study indicates that lactate accumulation in TME induces METTL3 upregulation in TANs via H3K18 lactylation, thereby enhancing the JAK1/STAT3 signaling pathway in an m^6^A-dependent manner and fostering an immunosuppressive CRC TME (88). Considering the critical role of TANs in TME, further in-depth investigation into this area is warranted.

#### 3.2.5 Cancer-associated fibroblasts

CAFs are located within or near tumors and originate from diverse cellular sources, including bone marrow mesenchymal stem cells, epithelial cells, endothelial cells, and adipocytes (101). They facilitate metabolic interactions among CAFs, cancer cells, and immune cells by activating glycolysis, glutamine metabolism, and lipid metabolism, producing metabolites such as lactate, aspartate, and lipids (102–104). CAF metabolic abnormalities in CRC are known to remodel TME and enhance CRC cell proliferation. During co-culture with colon cancer cells, CAFs display increased glycolytic activity (105), fatty acid and phospholipid accumulation (104), and heightened glutamine catabolism (102). This interaction via metabolites contributes to an immunosuppressive TME. Although the existing evidence on the crosstalk among CAFs, m^6^A modifications, and metabolites is incomplete, one study demonstrated that CAF-derived METTL3-containing exosomes can upregulate acyl-CoA synthetase long-chain family member 3 (ACSL3) expression in an m^6^A-dependent manner, thus inhibiting ferroptosis and promoting CRC metastasis (106). Given METTL3’s role in regulating various metabolites in TME, these metabolites may act as signaling molecules in m^6^A-mediated crosstalk between fibroblasts and tumor cells, necessitating further investigation.

## 4 Potential clinical applications of m^6^A and its metabolites in CRC

### 4.1 Potential clinical applications of m^6^A modifications and metabolites in CRC diagnosis, prognosis, and treatment response prediction

#### 4.1.1 Potential clinical applications of m^6^A modification in diagnosis, prognosis, and prediction of treatment response in CRC

To date, several studies have investigated the diagnostic potential of m^6^A modifications and regulators in CRC. METTL3-m^6^A enhances cancer cell proliferation, invasion, and resistance by regulating key signaling pathways in CRC, positioning METTL3 as a promising biomarker for diagnosis (107). In a certain study, peripheral blood immune cells from the CRC group exhibited significantly elevated m^6^A RNA levels compared with those from the normal control group (108). Further research has revealed that m^6^A RNA in these immune cells serves as a promising non-invasive diagnostic biomarker for CRC, surpassing the efficacy of carcinoembryonic antigen, cancer antigen 125, and carbohydrate antigen 19-9 (108). As a result, research on m^6^A modification—as an emerging cancer biomarker—not only deepens our understanding of CRC diagnosis but also holds significant potential for improving early detection.

In addition to diagnostic applications, m^6^A modifications and regulators are linked to CRC prognosis. In CRC cells, METTL3 stabilizes SRY-box transcription factor 2 expression via m^6^A-mediated IGF2BP2 recognition, suggesting a potential biomarker combination for CRC prognosis (109). Besides, researchers have identified METTL14 (110, 111), METTL16 (11), WTAP (112), FTO (113), and ALKBH5 (114) as determinants of poor prognosis in patients with CRC (11). Data from the Cancer Genome Atlas further confirm the critical role of m^6^A methylation regulators in CRC prognosis (115). Exciting advancements in bioinformatics have prompted the development of numerous software tools for cancer prediction. The bioinformatics tool RNAMethyPro employs a novel gene expression signature comprising seven m^6^A regulators to predict prognosis across diverse cancers (116). Another promising application of m^6^A modification is the prediction of responses to cancer treatments. Researchers have created a risk model called the m^6^A score through principal component analysis, which categorizes patients into high- and low-m^6^A-score groups (117). Further analysis indicates that the m^6^A score is a strong predictor of overall survival and the clinical efficacy of immunotherapy in patients with CRC (117).

Consequently, m^6^A regulators and m^6^A-modified RNAs potentially serve as valuable biomarkers, prognostic indicators, and therapeutic targets (118).

#### 4.1.2 Potential clinical applications of metabolites in the diagnosis, prognosis, and prediction of treatment response in CRC

Metabolic dysregulation is a hallmark of CRC, and metabolites linked to this dysregulation can accurately reflect the condition of tumor cells and tissues. Accordingly, metabolites offer potential for patient-specific diagnosis, prognostic assessment, and treatment outcome evaluation (**Table 1**).

**Table 1:** Diagnosis and prognosis of CRC based on metabolites.

Metabolites	Role of metabolites in CRC	Detection of specimen	Metabolic regulation	References
Oleic acid and isocitric	Early stage of CRC	Serum metabolites detection	Lipid metabolism and glucose metabolism	(119)
Phosphatidylglycerol, fatty acids, sterol esters, and ceramides	Progress of CRC	Serum metabolites detection	Lipid metabolism	(120)
Glycerophospholipids, sphingolipids, and caffeine	Advanced adenoma	Serum metabolites detection	Lipid metabolism	(121)
Benzoic acid, caprylic acid, decanoic acid, and histidine	CRC staging assessment	Serum metabolites detection	Gut microbiota metabolites, Lipid metabolism, and amino acid metabolism	(122)
Citrulline and histidine	CRC staging assessment	Serum metabolites detection	amino acid metabolism	(123–125)
Glycine, serine, and threonine	Predicting LARC response to NCRT	Serum metabolites detection	amino acid metabolism	(127)
3-(1H-indol-3-yl)-2-(trimethylammonio) propanoate, 3-(3-hydroxyphenyl)-3-hydroxypropionic acid, N-methyl-L-proline, trimethylsilyl ester, 3,4-dihydroxyhydrocinnamic acid NIST; hippuric acid, N-omega-acetylhistamine, and saccharin	Early detection of CRP	Urinary metabolites detection	Amino acid metabolism and its derivative metabolites, ut microbiota metabolites,	(128)
Hydroxyproline dipeptide, tyrosine, glucuronic acid, tryptophan, pseudouridine, glucose, glycine, histidine, 5-oxoprolinic acid, isocitric acid, and succinic acid, while citric acid, octadecanoic acid, hexadecanoic acid, and propanoic acid-2-methyl-1-(1,1-dimethylethyl)-2-methyl-1,3-propanediyl ester	Diagnosis of CRC	Urinary metabolites detection	Lipid metabolism, glucose metabolism , amino acid metabolism and their derivative metabolites	(129)
Millerol M, arachidonic acid, FAHFAs, and deglutamine	Diagnoses of NC, CRA and CRC	Fecal metabolites detection	Lipid metabolism, amino acid metabolism	(119)
Acetates, butyrates, propionates, 3-hydroxyphenylacetic acid, valine, tyrosine, and leucine	Diagnosis of CRC and AP	Fecal metabolites detection	Lipid metabolism, amino acid metabolism	(130)
Sphingolipids, and cholesterol esters	Diagnosis of CRC	Fecal metabolite detection	Lipid metabolism	(132)
Fatty acids	Diagnosis of CRC	Fecal metabolites detection	Lipid metabolism	(131)
Glucose, glutamate, alanine, valine, and histidine	Evaluation of CRC location and stage	Histological metabolite detection	Glucose metabolism, lipid metabolism and their derivatives	(133)
Glycine	Predicting treatment response	Histological metabolite detection	Amino acid metabolism	(134)
Lipids, amino acids, and choline	Evaluation of CRC staging	Histological metabolite detection	Lipid metabolism, amino acid metabolism	(135)
S. flexneri C.11 and agmatine	Diagnosis of CRC	Serum, fecal, and Histological metabolite detection	Gut microbiota metabolites, amino acid metabolism	(137)
IAA	Predicting treatment response	Fecal metabolites detection	Amino acid metabolism	(138)

Significant advances have been made in the utilization of blood metabolite detection for CRC diagnosis and prognosis. Plasma metabolites such as oleic and isocitric acids exhibit promise for the early detection of CRC (119), while lipid biomarkers—including phosphatidylglycerol, fatty acids, sterol esters, and ceramides—are associated with CRC metabolism (120). Further studies have revealed that serum metabolites in advanced adenoma resemble those in CRC, including glycerophospholipids, sphingolipids, and caffeine metabolism (121). This underscores the crucial role of serum metabolites in early CRC prediction and diagnosis. Additionally, researchers employing capillary electrophoresis–time-of-flight mass spectrometry, an innovative technique for detecting metabolites, discovered that the blood metabolite benzoic acid possesses excellent diagnostic potential and could serve as a novel biomarker for CRC (122). Moreover, correlations of benzoic acid, caprylic acid, decanoic acid, and histidine with CRC staging were observed (122). These findings suggest that blood metabolites contribute to diagnosis and significantly inform tumor staging. An international collaborative study involving 744 patients with CRC utilized serum metabolomics analysis with gas chromatography/mass spectrometry, revealing significantly lower plasma citrulline and histidine levels in stage IV patients compared with those in stage I patients (123). Notably, serum histidine levels were particularly reduced in metastatic CRC (124, 125). Blood metabolites can also predict treatment outcomes for CRC. Researchers have identified 17 potential biomarkers in plasma samples from patients with CRC that accurately forecast postoperative outcomes following tumor resection (126). Furthermore, non-targeted metabolomics analysis via liquid chromatography–mass spectrometry on serum samples from patients with locally advanced rectal cancer (LARC) undergoing neoadjuvant chemoradiotherapy (NCRT) indicated that metabolites such as glycine, serine, and threonine could predict LARC response to NCRT (127). This offers promise for developing personalized treatment strategies for patients with LARC.

Urinary metabolites have also displayed significant potential for CRC diagnosis. Xie Y et al. (128) identified seven urinary metabolites—3-(1H-indol-3-yl)-2-(trimethylammonio) propanoate, 3-(3-hydroxyphenyl)-3-hydroxypropionic acid, N-methyl-L-proline trimethylsilyl ester, 3,4-dihydroxyhydrocinnamic acid NIST, hippuric acid, N-omega-acetylhistamine, and saccharin—as promising non-invasive biomarkers for colorectal polyps, aiding early detection. Similarly, Ning et al. (129) identified 11 upregulated and 4 downregulated metabolites in urine samples from patients with CRC compared to those from healthy participants. Notable increases were observed in hydroxyproline dipeptide, tyrosine, glucuronic acid, tryptophan, pseudouridine, glucose, glycine, histidine, 5-oxoprolinic acid, isocitric acid, and succinic acid, whereas citric acid, octadecanoic acid, hexadecanoic acid, and propanoic acid-2-methyl-1-(1,1-dimethylethyl)-2-methyl-1,3-propanediyl ester decreased significantly (129).

Fecal metabolites also play an indispensable role in CRC diagnosis. Research has identified significant alterations in fecal metabolites at various stages of CRC progression. Specific metabolites—including Millerol M, arachidonic acid, fatty acyl esters of hydroxyl fatty acids, and deglutamine—vary among non-cancerous colitis, precancerous lesions, and CRC, following the adenoma-to-carcinoma sequence (119). Likewise, acetates, butyrates, propionates, 3-hydroxyphenylacetic acid, valine, tyrosine, and leucine exhibit significant differences in the fecal metabolomic profiles of patients with CRC, those with adenomatous polyposis, and healthy controls (130). Furthermore, fecal metabolites, such as fatty acids (131), sphingolipids, and cholesterol esters (132) in patients with CRC may also serve as screening tools. Collectively, these studies corroborate that fecal metabolites potentially act as non-invasive biomarkers for diagnosing CRC.

While liquid biopsy offers advantages, including ease of sampling, effective monitoring, and the longitudinal assessment of treatment dynamics, histological examination remains essential for CRC diagnosis and prognosis. Compared to left-sided colon cancer, right-sided colon cancer exhibits increased glucose and succinate levels alongside decreased inositol content (133). Elevated glycine concentrations (> 1.77 μmol/g) in tumor tissues correlate with poorer progression-free survival in patients with LARC and potentially predict treatment response (134). Furthermore, distinct metabolites—glucose, glutamate, alanine, valine, and histidine—differentiate CRC tissue (CCT) from distant non-cancerous tissue (DNT) (133). The combination of these five metabolites can also distinguish between CCT stages I/II and III/IV (133). Moreover, the metabolomic profiles of 50 human CCTs and adjacent non-cancerous tissues (ANITs) (135) revealed that metabolites such as lipids, amino acids, and choline differentiate CCTs from ANITs, as well as low-grade tumors (stages I–II) from high-grade tumors (stages III–IV) (135). Collectively, these studies reveal characteristic metabolomic phenotypes associated with CCT and highlight the importance of tissue metabolomics as a molecular–pathological tool for CRC diagnosis and prognosis.

Interestingly, gut bacteria produce metabolites such as secondary bile acids from primary bile acids, hydrogen sulfide, and trimethylamine-N-oxide, which potentially contribute to inflammation and cancer development (136). Accordingly, microbiota-derived metabolites could play a role in CRC diagnosis, prognosis, and treatment (136). For instance, the gut strain *Shigella flexneri* C.11 in patients with CRC upregulates Erb-B2 receptor tyrosine kinase 3, activating the PI3K/AKT pathway and promoting colitis-associated tumorigenesis (137). Clinically, co-detection of the *S. flexneri* C.11 strain and guanidine can differentiate patients with CRC from healthy controls, indicating its potential as a non-invasive diagnostic biomarker for CRC (137). Moreover, in patients with CRC undergoing clinical irinotecan therapy, levels of indole-3-acetic acid (IAA)—a tryptophan metabolite derived from *Bacteroides intestinalis*—strongly correlate with diarrhea severity, suggesting IAA as a potential predictive biomarker (138). These studies reveal the diagnostic and prognostic significance of gut microbiota characteristics and metabolites in patients with CRC.

### 4.2 The potential of targeting m^6^A modification and metabolites in CRC therapy

#### 4.2.1 Clinical application potential of targeting m^6^A modification

Research increasingly underscores the potential of m^6^A modification inhibitors as targeted anticancer therapies, with promising developments in CRC treatment (**Table 2**). STM2457, a METTL3 inhibitor, suppresses CRC progression through multiple mechanisms. Specifically, it stabilizes olfactory receptor family 51 subfamily E member 2 expression, inhibiting CRC cell proliferation (139). Additionally, an adhesive hemostatic hydrogel loaded with STM2457 enhances the expression of CXCL9 and CXCL10, thereby recruiting CAR-NK cells to eliminate tumor cells (72). The combination of this hydrogel with CAR-NK cell therapy significantly mitigates CRC recurrence *in vivo* (72). Despite developing several small-molecule inhibitors targeting m^6^A-modifying methyltransferases with proven antitumor efficacy, their application in CRC treatment remains limited. Considering that m^6^A modification reshapes cancer metabolic networks by regulating metabolic gene expression and metabolism-related signaling pathways, clinical research targeting this pathway presents significant potential for CRC development, warranting further exploration.

**Table 2:** CRC-targeted drug therapy based on m^6^A methylation.

Drug name	Targeting	Therapy mechanism	Reference
STM2457	METTL3/14	STM2457 stably expresses OR51E2 in a m^6^A-dependent manner via METTL3/14, thereby inhibiting CRC cell proliferation.	(139)
STM2457	METTL3	An adhesive hemostasis hydrogel loaded with STM2457 enhances the expression of CXCL9 and CXCL10, thereby recruiting CAR-NK cells to induce tumor cell killing.	(72)
GA	METTL3	GA downregulates METTL3 expression, thereby reducing p62 mRNA stability in an m^6^A-dependent manner and inducing ferroptosis in tumor cells	(159)

#### 4.2.2 Clinical application potential of targeting metabolites

The abnormal expression of intracellular metabolic enzymes and the accumulation of related metabolites potentially trigger carcinogenic signals and promote tumorigenesis. Consequently, drugs targeting metabolic enzymes and associated metabolites are emerging as promising strategies for CRC treatment (**Table 3**).

**Table 3:** CRC-targeted drug therapy based on related metabolic enzymes/metabolites.

Drug name	Related metabolic enzymes	Functions	References
GB2	TREM2	GB2 induces metabolic reprogramming by targeting the TREM2 receptor on their surface in TAMs, thereby inducing them to exhibit a pro-inflammatory M1 phenotype	(140)
Worenine, Berberine Matrine, Quercetin Rosmarinic acid	HIF-1α	Worenine, Berberine, Matrine, Quercetin, and Rosmarinic acid inhibit glycolysis by degrading HIF-1α via ubiquitination, thereby suppressing tumor progression	(141–143) (144, 145)
DHP-B	CPT1A	specifically inhibits CPT1A, thereby blocking mitochondrial β-oxidation and inducing CRC cell apoptosis	(160)
LCL-521	ASAH1	LCL-521 activates M1 macrophages and enhances cytotoxic T cell infiltration, thereby establishing an anti-tumor immune microenvironment	(148)
Ketogenic diet	Lipid metabolism	Generating stearic acid to inhibit CRC growth by inducing Th17 cell differentiation	(149)
Atorvastatin	Lipid metabolism	Statin atorvastatin induces apoptosis in CRC cells by causing mitochondrial dysfunction	(161)
GQD	GLS1	GQD can significantly inhibit CRC xenograft growth in nude mice by specifically downregulating GLS1 expression via the YTHDF1/m6A axis	(45)
Poliumoside	GPT1	poliumoside inhibits tumorigenesis through dual mechanisms: α-KG-mediated WNT signaling suppression and disruption of the folate cycle	(162)
MAG	Kyn/Trp	MAG inhibits CRC growth by influencing gut microbiota-tryptophan metabolism, thereby reducing the Kyn/Trp ratio	(57)
JPJDF	Trp	JPJDF improves intestinal barrier function by regulating the tryptophan metabolism-AhR pathway, thereby reducing MDSC infiltration to promote the formation of an anti-tumor immune microenvironment	(58)
ZQFZ	SCFA	ZQFZ can reshape gut microbiota composition, thereby increasing SCFA levels, which promotes M1 macrophage polarization and CD8^+^ T cell infiltration.	(56)

Targeting glycolytic metabolites and related metabolic enzymes

Innovative research is increasingly focusing on metabolic regulation within TME. Recent findings indicate that the stimulator of interferon genes (STING) agonist prodrug GABAB receptor subunit 2 induces metabolic reprogramming by targeting the “triggering receptor expressed on myeloid cells 2 (TREM2)” receptor on TAMs. This activation of the STING signaling pathway encourages a pro-inflammatory M1 phenotype (140). This dual immunometabolic regulatory strategy reveals new possibilities for tumor immunotherapy research. Not only that, but some natural compounds have shown promise in treating CRC by targeting the hypoxic TME and glucose metabolism. HIF-1α is a transcription factor that is highly sensitive to oxygen. Worenine (141), berberine (142), matrine (143), quercetin (144), and rosmarinic acid (145), inhibit glycolysis by promoting HIF-1α degradation via ubiquitination, further suppressing tumor progression. These studies underline the significant role of phytochemical drugs in CRC treatment. Future research should prioritize synergistic therapies that integrate natural compounds with chemotherapy or targeted drugs to enhance the clinical application.

Targeting lipid metabolites and related metabolic enzymes

Targeting lipid metabolites and related metabolic enzymes in CRC treatment involves various regulatory mechanisms, with recent research revealing innovative strategies for tumor therapy. Current research indicates that lipid metabolic enzyme inhibitors can influence the hypoxic TME and tumor angiogenesis by interfering with pivotal metabolic pathways—such as fatty acid synthesis, oxidation, and membrane lipid remodeling, thereby significantly inhibiting the proliferation and survival of CRC cells. For example, Oroxylin A reduces intracellular fatty acid levels and enhances FAO by inhibiting FASN and sterol regulatory element-binding protein 1 (SREBP1) through the downregulation of HIF-1α, consequently suppressing tumor progression (146). Furthermore, RO48–8071 hinders CRC angiogenesis and metastatic potential by inhibiting oxidosqualene cyclase (OSC), a key enzyme in cholesterol biosynthesis (147).

Comprehensive studies have revealed that certain lipid metabolism enzymes interact dynamically with immune cells. LCL-521, an inhibitor of the sphingolipid metabolism enzyme acidic ceramidase (ASAH1), activates M1 macrophages and enhances cytotoxic T cell infiltration by inducing type I and II interferon responses, thus creating an antitumor immune microenvironment (148). Gut microbiota metabolites also substantially contribute to TME remodeling. The ketogenic diet, which substitutes carbohydrates with fats as the primary energy source, generates stearic acid to inhibit CRC growth by inducing Th17 cell differentiation (149). Its metabolic intermediate, β-hydroxybutyrate, modulates TAN polarization via the HDAC3–AMOT–YAP/TAZ axis, further fostering an antitumor immune microenvironment (150). These findings suggest that targeting lipid metabolism not only directly suppresses tumor cell growth but also elicits systemic antitumor effects by reconfiguring TME.

These findings suggest that integrating lipid metabolism modulators with conventional chemotherapy or targeted therapies could address resistance issues associated with monotherapy, paving the way for personalized CRC treatment. Future research should explore the interactions among lipid metabolic enzymes, the microbiome, and the immune system, aiming to develop multi-omics data-based precision-targeting strategies that maximize therapeutic efficacy.

Targeting amino acid metabolites and related metabolic enzymes

Targeting amino acid metabolites and their associated metabolic enzymes presents innovative therapeutic strategies for CRC. CRC cells exhibit a metabolic dependence on glutamine, and the GLS inhibitor CB-839 suppresses tumor cell proliferation by inhibiting GLS activity, leading to intracellular glutamine accumulation and glutamate depletion (151). Additionally, Gegen Qinlian Decoction (GQD), a traditional Chinese herbal formula, significantly inhibits CRC xenograft growth in nude mice by selectively downregulating GLS1 expression through the YTHDF1/m6A axis (45).

Exogenous amino acid regulation serves an essential role in preventing and treating CRC. Exogenous serine deprivation leads to the upregulation of Forkhead box C1 expression by activating the ERK1/2–p-ELK1 signaling axis, establishing a negative feedback loop that promotes serine synthesis (152). Targeting this axis alongside a serine-restricted diet may innovatively enhance the effectiveness of 5-FU therapy (152). Moreover, animal studies demonstrate that a serine/glycine-free diet (-SG diet) not only hinders tumor growth but also promotes cytotoxic T cell infiltration, thus improving the antitumor immune microenvironment (153). A clinical trial (ChiCTR2300067929) has preliminarily confirmed the safety and immunomodulatory potential of the -SG diet (153). Additionally, methionine restriction reportedly enhances PD-1 blockade efficacy by suppressing proprotein convertase subtilisin/kexin type 9 (PCSK9) expression, which facilitates CD8^+^ T cell infiltration into TME and amplifies antitumor immune responses (154).

The gut microbiota and its metabolites are also pivotal in CRC development. Low dietary intake of branched-chain amino acids (BCAAs) partially counteracts CRC cell proliferation induced by *Clostridium symbiosum* (155), highlighting the synergistic effects of nutritional interventions and microbial regulation. In addition, magnolol (MAG), a natural lignan, inhibits CRC growth by affecting gut microbiota and tryptophan metabolism, leading to a reduced kynurenine (Kyn)/tryptophan (Trp) ratio (57). Research indicates that modulating gut microbiota can influence TME to suppress tumor progression. For instance, the traditional Chinese medicine Jianpi Jiedu Decoction enhances intestinal barrier function by regulating the tryptophan metabolism–aryl hydrocarbon receptor pathway through gut microbiota modulation, thereby decreasing MDSC infiltration and fostering an antitumor immune microenvironment (58). Oral administration of a nanomedicine-engineered bacterium (LR-S-CD/CpG@LNPs) elevates indole-3-carboxaldehyde levels by stimulating *Limosilactobacillus reuteri* to degrade tryptophan, thus augmenting the immunotherapeutic response to *in situ* CRC and inhibiting metastasis (156). Notably, a high-salt diet can diminish the efficacy of FOLFOX therapy for CRC through macrophage-mediated immunomodulatory mechanisms by altering gut microbiota and tryptophan metabolism (59), accentuating the importance of low-salt dietary management in optimizing treatment. Additionally, Zhenqi Fuzheng Granules can reshape gut microbiota composition, thereby increasing SCFA levels. This phenomenon promotes M1 macrophage polarization and CD8^+^ T cell infiltration, thus remodeling CRC immune microenvironment (56).

The combined use of metabolic-enzyme and metabolite targeting alongside chemotherapy or targeted therapies displays significant clinical potential. The indoleamine 2,3-dioxygenase inhibitor epacadostat markedly reduced the migration and proliferation of cetuximab-resistant CRC cells by inhibiting tryptophan metabolism, leading to a decreased Kyn/Trp ratio (157). Further research revealed a notable synergistic effect when cetuximab was used in conjunction with epacadostat to combat the resistance (157). Remarkably, synergistic combinations between gut microbiota-specific metabolites and certain agents, such as MAG, have gained attention for their ability to enhance antitumor effects (57). These findings imply that multidimensional strategies integrating metabolic regulation, dietary intervention, and microbial modulation could lead to more precise and effective CRC treatments.

### 4.3 Potential clinical applications of combining targeted m^6^A modification with metabolite-based therapy in tumors

As previously noted, m^6^A modification in CRC is closely linked to metabolic reprogramming and metabolites. Strategies targeting m^6^A modifications, metabolic enzymes, or metabolites have demonstrated clinical utility. Nonetheless, existing monotherapies often fail to achieve optimal efficacy and may cause varying degrees of toxicity. Consequently, multifaceted combination therapy is emerging as a crucial focus in modern cancer treatment. Considering the complex interplay among m^6^A modification, metabolic regulatory networks, and TME, targeting m^6^A modification and key metabolic pathways simultaneously may generate synergistic antitumor effects. This represents a considerably promising therapeutic approach that could significantly improve treatment outcomes. Nevertheless, systematic research on this topic in CRC remains limited, and clinical translation has yet to yield definitive conclusions (**Table 4**).

**Table 4:** The potential combined targeted therapy of m^6^A enzymes and related metabolic enzymes/metabolites.

Targeting	Tardeted related metabolic enzymes/metabolites	Combination therapy mechanism	References
METTL3	LDHA	Combined inhibition of METTL3 and LDHA enhances sensitivity to 5-FU chemotherapy in drug- resistant CRC patients	(20)
YTHDF1	GLS1	Combined inhibition of YTHDF1 and GLS1 synergistically restored cisplatin sensitivity in cisplatin-resistant colon cancer cells	(14)
YTHDF1	GLS1	Simultaneous inhibition of YTHDF1 and GLS1 expression exhibits potent synergistic antitumor effects	(45)
YTHDF1	Met	MRD and YTHDF1 depletion facilitates antitumor immunity	(63)

METTL3 has been identified as a functional and clinically oncogenic gene in CRC. Inhibiting METTL3 attenuates the Warburg effect, thereby hindering tumor progression (22, 158). Concurrently, LDHA is overexpressed in CRC, facilitating the conversion of pyruvate to lactate, which triggers glycolysis and 5-FU resistance (19, 20). Therefore, LDHA is widely recognized as a therapeutic target in cancer treatment. Considering these insights, a rational combination therapy strategy targeting METTL3 and glycolysis-related processes is warranted for CRC characterized by elevated glucose metabolism. Progress has been made, as Zhang et al. (20) demonstrated that the combined inhibition of METTL3 and LDHA significantly improves *in vivo* sensitivity to 5-FU chemotherapy in drug-resistant patients with CRC. In the context of amino acid metabolism, YTHDF1 promotes GLS1 synthesis in an m^6^A-dependent manner, with GLS1 being a key enzyme in glutamine metabolism (14). The concurrent inhibition of YTHDF1 and GLS1 has proven to synergistically restore cisplatin sensitivity in cisplatin-resistant colon cancer cells (14). Similarly, GQD inhibits GLS1 expression via YTHDF1 in an m^6^A-dependent manner, reducing tumor volume in nude mouse xenografts (45). Notably, the simultaneous inhibition of YTHDF1 and GLS1 expression exerts potent synergistic antitumor effects (45). Additionally, methionine-restricted diets or YTHDF1 depletion can suppress tumor growth by restoring CD8^+^ T cell infiltration and synergize with PD-1 blockade for improved tumor control (63). These studies provide a theoretical basis for concurrently targeting m^6^A modification and metabolic pathways, rendering this a promising therapeutic strategy despite the current lack of clinical research.

## 5 Conclusions and outlook

Metabolic reprogramming is a defining characteristic of CRC. In CRC, Multiple metabolic pathways frequently become dysregulated, leading to abnormal glucose, lipid, and amino acid metabolism. These altered metabolic processes supply tumor cells with the energy, nutrients, and redox conditions necessary for malignant growth and metastasis while concomitantly impairing metabolic activity in TME and contributing to gut microbiota dysbiosis. Recent studies have highlighted the extensive role of m^6^A modification in CRC-related metabolic pathways, significantly influencing tumor progression. Notably, m^6^A modification plays a central role in regulating metabolite levels associated with glucose, amino acid, and lipid metabolism. The dysregulated accumulation of these metabolites potentially activates oncogenic signaling pathways, promoting tumorigenesis. Furthermore, the crosstalk between m^6^A modification and metabolic reprogramming not only affects tumor cells but also has profound implications for TME.

Although progress has been made in understanding how m^6^A modifications affect TME, the mechanism by which m^6^A modifications regulate the immunosuppressive TME in CRC is still mainly based on cell data studies, with scarce specific causal evidence. Further in-depth research is necessary to determine how m^6^A modification contributes to the formation of an immunosuppressive TME in CRC. Current insights suggest that a network involving m^6^A modification, metabolic reprogramming, and TME significantly enhances CRC cell proliferation, metastasis, and drug resistance. Therefore, targeting m^6^A modifications, metabolic pathways, and immune cell populations—either individually or in combination—holds considerable potential for improving CRC treatment efficacy.

Currently, inhibitors that target m^6^A modification enzymes or metabolic pathways have demonstrated clinical efficacy. However, their effectiveness is limited by suboptimal inhibition efficiency, toxicity, and resistance. Consequently, combined targeted therapies that address both m^6^A modification and metabolic pathways may overcome these limitations. Notably, promising outcomes from the combined use of m^6^A modification and metabolite therapies have been reported. Notwithstanding, further research is warranted to validate their clinical potential and therapeutic efficacy. Based on the insights from the m^6^A–metabolic reprogramming–TME network discussed in this review, we can reasonably infer that combination strategies that target m^6^A modification and metabolic pathways may significantly improve treatment outcomes in CRC.

Overall, a comprehensive investigation of the metabolic network surrounding the m^6^A–metabolic reprogramming–TME axis in CRC, along with an understanding of how these interactions influence tumor biology and immune cell function, presents significant clinical promise.

## References

[1] Sung H Ferlay J Siegel RL Laversanne M Soerjomataram I Jemal A . Global cancer statistics 2020: GLOBOCAN estimates of incidence and mortality worldwide for 36 cancers in 185 countries. CA Cancer J Clin. (2021) 71:209–49. doi: 10.3322/caac.21660 33538338

[2] Chen B Hong Y Gui R Zheng H Tian S Zhai X . N6-methyladenosine modification of circ_0003215 suppresses the pentose phosphate pathway and Malignancy of colorectal cancer through the miR-663b/DLG4/G6PD axis. Cell Death Dis. (2022) 13:804. doi: 10.1038/s41419-022-05245-2 36127319 PMC9489788

[3] Dekker E Tanis PJ Vleugels JLA Kasi PM Wallace MB . Colorectal cancer. Lancet. (2019) 394:1467–80. doi: 10.1016/S0140-6736(19)32319-0 31631858

[4] Hanahan D Weinberg RA . Hallmarks of cancer: the next generation. Cell. (2011) 144:646–74. doi: 10.1016/j.cell.2011.02.013 21376230

[5] An Y Duan H . The role of m6A RNA methylation in cancer metabolism. Mol Cancer. (2022) 21:14. doi: 10.1186/s12943-022-01500-4 35022030 PMC8753874

[6] Ge H Yan Y Wang H Bian J Deng Z Su X . Circular RNA hsa_circ_0005939 regulates UHRF1BP1L expression by targeting miR-4693-3p to promote colorectal cancer progression. Protein Pept Lett. (2024) 31:437–46. doi: 10.2174/0109298665297110240611115010 38918974

[7] Tian S Chen M Jing W Meng Q Wu J . miR-1204 positioning in 8q24.21 involved in the tumorigenesis of colorectal cancer by targeting MASPIN. Protein Pept Lett. (2024) 31:544–58. doi: 10.2174/0109298665305114240718072029 39082173

[8] Wang T Kong S Tao M Ju S . The potential role of RNA N6-methyladenosine in cancer progression. Mol Cancer. (2020) 19:88. doi: 10.1186/s12943-020-01204-7 32398132 PMC7216508

[9] Huang J Shao Y Gu W . Function and clinical significance of N6-methyladenosine in digestive system tumours. Exp Hematol Oncol. (2021) 10:40. doi: 10.1186/s40164-021-00234-1 34246319 PMC8272376

[10] Han X Wang L Han Q . Advances in the role of m(6)A RNA modification in cancer metabolic reprogramming. Cell Biosci. (2020) 10:117. doi: 10.1186/s13578-020-00479-z 33062255 PMC7552565

[11] Wei W Zhang ZY Shi B Cai Y Zhang HS Sun CL . METTL16 promotes glycolytic metabolism reprogramming and colorectal cancer progression. J Exp Clin Cancer Res. (2023) 42:151. doi: 10.1186/s13046-023-02732-y 37340443 PMC10280857

[12] Li N Kang Y Wang L Huff S Tang R Hui H . ALKBH5 regulates anti-PD-1 therapy response by modulating lactate and suppressive immune cell accumulation in tumor microenvironment. Proc Natl Acad Sci USA. (2020) 117:20159–70. doi: 10.1073/pnas.1918986117 32747553 PMC7443867

[13] Zhang Q Du Z Zhou W Li W Yang Q Yu H . ZDHHC1 downregulates LIPG and inhibits colorectal cancer growth via IGF2BP1 palmitoylation. Cancer Gene Ther. (2024) 31:1427–37. doi: 10.1038/s41417-024-00808-1 39069526 PMC11405259

[14] Chen P Liu XQ Lin X Gao LY Zhang S Huang X . Targeting YTHDF1 effectively re-sensitizes cisplatin-resistant colon cancer cells by modulating GLS-mediated glutamine metabolism. Mol Ther Oncolytics. (2021) 20:228–39. doi: 10.1016/j.omto.2021.01.001 33614908 PMC7873577

[15] Zhao L Guo J Xu S Duan M Liu B Zhao H . Abnormal changes in metabolites caused by m(6)A methylation modification: the leading factors that induce the formation of immunosuppressive tumor microenvironment and their promising potential for clinical application. J Adv Res. (2025) 70:159–86. doi: 10.1016/j.jare.2024.04.016 38677545 PMC11976433

[16] Abi Zamer B Abumustafa W Hamad M Maghazachi AA Muhammad JS . Genetic mutations and non-coding RNA-based epigenetic alterations mediating the Warburg effect in colorectal carcinogenesis. Biol (Basel). (2021) 10:847. doi: 10.3390/biology10090847 34571724 PMC8472255

[17] La Vecchia S Sebastian C . Metabolic pathways regulating colorectal cancer initiation and progression. Semin Cell Dev Biol. (2020) 98:63–70. doi: 10.1016/j.semcdb.2019.05.018 31129171

[18] Zhou M He J Li Y Jiang L Ran J Wang C . N(6)-methyladenosine modification of REG1alpha facilitates colorectal cancer progression via beta-catenin/MYC/LDHA axis mediated glycolytic reprogramming. Cell Death Dis. (2023) 14:557. doi: 10.1038/s41419-023-06067-6 37626036 PMC10457312

[19] Wang C Liang W Zhong J Liu J Zhou C Ma C . m6A-mediated regulation of CPSF6 by METTL3 promotes oxaliplatin resistance in colorectal cancer through enhanced glycolysis. Cell Signal. (2025) 130:111676. doi: 10.1016/j.cellsig.2025.111676 40010558

[20] Zhang K Zhang T Yang Y Tu W Huang H Wang Y . N(6)-methyladenosine-mediated LDHA induction potentiates chemoresistance of colorectal cancer cells through metabolic reprogramming. Theranostics. (2022) 12:4802–17. doi: 10.7150/thno.73746 35832094 PMC9254245

[21] Chen H Gao S Liu W Wong CC Wu J Wu J . RNA N(6)-methyladenosine methyltransferase METTL3 facilitates colorectal cancer by activating the m(6)A-GLUT1-mTORC1 axis and is a therapeutic target. Gastroenterology. (2021) 160:1284–300:e16. doi: 10.1053/j.gastro.2020.11.013 33217448

[22] Shen C Xuan B Yan T Ma Y Xu P Tian X . m(6)A-dependent glycolysis enhances colorectal cancer progression. Mol Cancer. (2020) 19:72. doi: 10.1186/s12943-020-01190-w 32245489 PMC7118901

[23] Li L Zhang R Li Y . Up-regulation of m(6)A writer METTL14 inhibits tumorigenesis by suppressing glycolysis in colorectal cancer. BMC Cancer. (2025) 25:305. doi: 10.1186/s12885-025-13532-2 39979838 PMC11844156

[24] Hou Y Zhang X Yao H Hou L Zhang Q Tao E . METTL14 modulates glycolysis to inhibit colorectal tumorigenesis in p53-wild-type cells. EMBO Rep. (2023) 24:e56325. doi: 10.15252/embr.202256325 36794620 PMC10074077

[25] Wang J Zhu M Zhu J Li J Zhu X Wang K . HES1 promotes aerobic glycolysis and cancer progression of colorectal cancer via IGF2BP2-mediated GLUT1 m6A modification. Cell Death Discov. (2023) 9:411. doi: 10.1038/s41420-023-01707-4 37957183 PMC10643658

[26] Wang Y Lu JH Wu QN Jin Y Wang DS Chen YX . LncRNA LINRIS stabilizes IGF2BP2 and promotes the aerobic glycolysis in colorectal cancer. Mol Cancer. (2019) 18:174. doi: 10.1186/s12943-019-1105-0 31791342 PMC6886219

[27] Lu S Han L Hu X Sun T Xu D Li Y . N6-methyladenosine reader IMP2 stabilizes the ZFAS1/OLA1 axis and activates the Warburg effect: implication in colorectal cancer. J Hematol Oncol. (2021) 14:188. doi: 10.1186/s13045-021-01204-0 34743750 PMC8574039

[28] Ye M Chen J Lu F Zhao M Wu S Hu C . Down-regulated FTO and ALKBH5 co-operatively activates FOXO signaling through m6A methylation modification in HK2 mRNA mediated by IGF2BP2 to enhance glycolysis in colorectal cancer. Cell Biosci. (2023) 13:148. doi: 10.1186/s13578-023-01100-9 37580808 PMC10424385

[29] Wu S Yun J Tang W Familiari G Relucenti M Wu J . Therapeutic m(6)A eraser ALKBH5 mRNA-loaded exosome-liposome hybrid nanoparticles inhibit progression of colorectal cancer in preclinical tumor models. ACS Nano. (2023) 17:11838–54. doi: 10.1021/acsnano.3c03050 37310898

[30] Chen D Zhou X Yan P Yang C Li Y Han L . Lipid metabolism reprogramming in colorectal cancer. J Cell Biochem. (2023) 124:3–16. doi: 10.1002/jcb.30347 36334309

[31] Pakiet A Kobiela J Stepnowski P Sledzinski T Mika A . Changes in lipids composition and metabolism in colorectal cancer: a review. Lipids Health Dis. (2019) 18:29. doi: 10.1186/s12944-019-0977-8 30684960 PMC6347819

[32] Chang L Ding J Pu J Zhu J Zhou X Luo Q . A novel lncRNA LOC101928222 promotes colorectal cancer angiogenesis by stabilizing HMGCS2 mRNA and increasing cholesterol synthesis. J Exp Clin Cancer Res. (2024) 43:185. doi: 10.1186/s13046-024-03095-8 38965575 PMC11223299

[33] Gao H Zheng S Liang J Wang Y Chen L Li H . m6A-induced DEAD-box RNA helicase 21 enhances lipid metabolism via 3-hydroxy-3-methylglutaryl-CoA synthases 1 in colorectal cancer. Transl Oncol. (2025) 55:102373. doi: 10.1016/j.tranon.2025.102373 40127603 PMC11979938

[34] Jiang T Qi J Xue Z Liu B Liu J Hu Q . The m(6)A modification mediated-lncRNA POU6F2-AS1 reprograms fatty acid metabolism and facilitates the growth of colorectal cancer via upregulation of FASN. Mol Cancer. (2024) 23:55. doi: 10.1186/s12943-024-01962-8 38491348 PMC10943897

[35] Li S Du M Xu K Ben S Zhu T Guo M . Genetic modulation of BET1L confers colorectal cancer susceptibility by reducing miRNA binding and m6A modification. Cancer Res. (2023) 83:2142–54. doi: 10.1158/0008-5472.CAN-22-0065 37115853

[36] Villar VH Merhi F Djavaheri-Mergny M Duran RV . Glutaminolysis and autophagy in cancer. Autophagy. (2015) 11:1198–208. doi: 10.1080/15548627.2015.1053680 26054373 PMC4590661

[37] Yang L Venneti S Nagrath D . Glutaminolysis: a hallmark of cancer metabolism. Annu Rev BioMed Eng. (2017) 19:163–94. doi: 10.1146/annurev-bioeng-071516-044546 28301735

[38] DeBerardinis RJ Lum JJ Hatzivassiliou G Thompson CB . The biology of cancer: metabolic reprogramming fuels cell growth and proliferation. Cell Metab. (2008) 7:11–20. doi: 10.1016/j.cmet.2007.10.002 18177721

[39] Peng W Zeng Z . Epigenetic activation of PTCD3 promotes CRC glutamine metabolism and metastasis via IGF2BP2-mediated SLC38A2 m6A modification. FASEB J. (2025) 39:e70558. doi: 10.1096/fj.202401788RR 40304977

[40] Song P Feng L Li J Dai D Zhu L Wang C . beta-catenin represses miR455-3p to stimulate m6A modification of HSF1 mRNA and promote its translation in colorectal cancer. Mol Cancer. (2020) 19:129. doi: 10.1186/s12943-020-01244-z 32838807 PMC7446108

[41] Han Y Pu Y Liu X Liu Z Chen Y Tang L . YTHDF1 regulates GID8-mediated glutamine metabolism to promote colorectal cancer progression in m6A-dependent manner. Cancer Lett. (2024) 601:217186. doi: 10.1016/j.canlet.2024.217186 39151722

[42] Han S Zhu L Zhu Y Meng Y Li J Song P . Targeting ATF4-dependent pro-survival autophagy to synergize glutaminolysis inhibition. Theranostics. (2021) 11:8464–79. doi: 10.7150/thno.60028 34373753 PMC8343999

[43] Qiao Y Su M Zhao H Liu H Wang C Dai X . Targeting FTO induces colorectal cancer ferroptotic cell death by decreasing SLC7A11/GPX4 expression. J Exp Clin Cancer Res. (2024) 43:108. doi: 10.1186/s13046-024-03032-9 38600610 PMC11005233

[44] Wang F Sun Z Zhang Q Yang H Yang G Yang Q . Curdione induces ferroptosis mediated by m6A methylation via METTL14 and YTHDF2 in colorectal cancer. Chin Med. (2023) 18:122. doi: 10.1186/s13020-023-00820-x 37735401 PMC10512537

[45] Lin X Xu L Gu M Shao H Yao L Huang X . Gegen Qinlian decoction reverses oxaliplatin resistance in colorectal cancer by inhibiting YTHDF1-regulated m6A modification of GLS1. Phytomedicine. (2024) 133:155906. doi: 10.1016/j.phymed.2024.155906 39089089

[46] Li Y Yang Y Zhang X Huang Z Zhang X Wen JJV . Colorectal cancer landscape from gut microbiota: insights into mutations, epigenetic dysregulation, and immune microenvironment alterations. (2026) 17:2696642. doi: 10.1080/21505594.2026.2696642 PMC13360637 42433082

[47] Nie K Yi J Yang Y Deng M Yang Y Wang T . A broad m6A modification landscape in inflammatory bowel disease. Front Cell Dev Biol. (2021) 9:782636. doi: 10.3389/fcell.2021.782636 35127705 PMC8809481

[48] Luo J Yu J Peng X . Could partial nonstarch polysaccharides ameliorate cancer by altering m(6)A RNA methylation in hosts through intestinal microbiota? Crit Rev Food Sci Nutr. (2022) 62:8319–34. doi: 10.1080/10408398.2021.1927975 34036843

[49] Zhu W Si Y Xu J Lin Y Wang JZ Cao M . Methyltransferase like 3 promotes colorectal cancer proliferation by stabilizing CCNE1 mRNA in an m6A-dependent manner. J Cell Mol Med. (2020) 24:3521–33. doi: 10.1111/jcmm.15042 32039568 PMC7131945

[50] Chen S Zhang L Li M Zhang Y Sun M Wang L . Fusobacterium nucleatum reduces METTL3-mediated m(6)A modification and contributes to colorectal cancer metastasis. Nat Commun. (2022) 13:1248. doi: 10.1038/s41467-022-28913-5 35273176 PMC8913623

[51] Zhou Z Niu Y Ma Y Zhang D Wang Y Ji R . Lipid accumulation inhibition strategies alleviate Fusobacterium nucleatum-infected colorectal cancer. Microbiome. (2025) 13:181. doi: 10.1186/s40168-025-02133-7 40770383 PMC12326762

[52] Cheng Y Wang Y Cheng Y Yang Q Zhang L Li Z . FOXD3-induced miR-133a blocks progression and metastasis of colorectal cancer through regulating UBA2. J Cancer. (2021) 12:6145–54. doi: 10.7150/jca.60647 34539887 PMC8425194

[53] Wong SH Yu J . Gut microbiota in colorectal cancer: mechanisms of action and clinical applications. Nat Rev Gastroenterol Hepatol. (2019) 16:690–704. doi: 10.1038/s41575-019-0209-8 31554963

[54] Krautkramer KA Fan J Bäckhed F . Gut microbial metabolites as multi-kingdom intermediates. Nat Rev Microbiol. (2021) 19:77–94. doi: 10.1038/s41579-020-0438-4 32968241

[55] O'Keefe SJ . Diet, microorganisms and their metabolites, and colon cancer. Nat Rev Gastroenterol Hepatol. (2016) 13:691–706. doi: 10.1038/nrgastro.2016.165 27848961 PMC6312102

[56] Guo L Yi J Zhang A Zheng X Wang M Yang F . Zhenqi Fuzheng Granule targets the SCFAs-GPR109A axis to enhance PD-1 antibody efficacy via immunometabolic remodeling in colorectal cancer. Phytomedicine. (2025) 148:157312. doi: 10.1016/j.phymed.2025.157312 41038145

[57] Ji H Qiao O Zhang Y Wang W Han X Zhang X . Dual targeting of wild-type p53 and gut microbiota by Magnolol represses key metabolic process and kills CRC cells. Phytother Res. (2024) 38:4982–98. doi: 10.1002/ptr.7924 37326338

[58] Chang Y Ou Q Zhou X Nie K Zheng P Liu J . Jianpi Jiedu decoction suppresses colorectal cancer growth by inhibiting M2 polarization of TAMs through the tryptophan metabolism-AhR pathway. Int Immunopharmacol. (2024) 138:112610. doi: 10.1016/j.intimp.2024.112610 38963982

[59] Deng Y Hou X Fang Q Wang H Li X Hu Z . High-salt diet decreases FOLFOX efficacy via gut bacterial tryptophan metabolism in colorectal cancer. Mol Med. (2025) 31:66. doi: 10.1186/s10020-025-01122-8 39972411 PMC11841010

[60] Xu W Zhou B Wang P Ma Y Jiang Y Mo D . N6-methyladenosine modification of 3'tRF-AlaAGC impairs PD-1 blockade efficacy by promoting lactic acid accumulation in the tumor microenvironment of gastric carcinoma. Drug Resist Update. (2025) 79:101197. doi: 10.1016/j.drup.2024.101197 39752904

[61] Yu A Fu L Jing L Wang Y Ma Z Zhou X . Methionine-driven YTHDF1 expression facilitates bladder cancer progression by attenuating RIG-I-modulated immune responses and enhancing the eIF5B-PD-L1 axis. Cell Death Differ. (2025) 32:776–91. doi: 10.1038/s41418-024-01434-y 39672819 PMC11982326

[62] Pan Y Chen H Zhang X Liu W Ding Y Huang D . METTL3 drives NAFLD-related hepatocellular carcinoma and is a therapeutic target for boosting immunotherapy. Cell Rep Med. (2023) 4:101144. doi: 10.1016/j.xcrm.2023.101144 37586322 PMC10439254

[63] Li T Tan YT Chen YX Zheng XJ Wang W Liao K . Methionine deficiency facilitates antitumour immunity by altering m(6)A methylation of immune checkpoint transcripts. Gut. (2022) 72:501–11. doi: 10.1136/gutjnl-2022-326928 35803704 PMC9933173

[64] Liu J Zhang W Chen L Wang X Mao X Wu Z . VSIG4 promotes tumour-associated macrophage M2 polarization and immune escape in colorectal cancer via fatty acid oxidation pathway. Clin Transl Med. (2025) 15:e70340. doi: 10.1002/ctm2.70340 40405491 PMC12098961

[65] Ippolito L Morandi A Giannoni E Chiarugi P . Lactate: A metabolic driver in the tumour landscape. Trends Biochem Sci. (2019) 44:153–66. doi: 10.1016/j.tibs.2018.10.011 30473428

[66] Brand A Singer K Koehl GE Kolitzus M Schoenhammer G Thiel A . LDHA-associated lactic acid production blunts tumor immunosurveillance by T and NK cells. Cell Metab. (2016) 24:657–71. doi: 10.1016/j.cmet.2016.08.011 27641098

[67] Liu Z Zheng N Li J Li C Zheng D Jiang X . N6-methyladenosine-modified circular RNA QSOX1 promotes colorectal cancer resistance to anti-CTLA-4 therapy through induction of intratumoral regulatory T cells. Drug Resist Update. (2022) 65:100886. doi: 10.1016/j.drup.2022.100886 36370665

[68] Huang X Sun T Wang J Hong X Chen H Yan T . Metformin reprograms tryptophan metabolism to stimulate CD8+ T-cell function in colorectal cancer. Cancer Res. (2023) 83:2358–71. doi: 10.1158/0008-5472.CAN-22-3042 37195082

[69] Zhang Y Zhao W Wu L Ai T He J Chen Z . Inhibition of CCT5-mediated asparagine biosynthesis and anti-PD-L1 produce synergistic antitumor effects in colorectal cancer. Acta Pharm Sin B. (2025) 15:2480–97. doi: 10.1016/j.apsb.2025.03.026 40487665 PMC12144965

[70] Yu T Van der Jeught K Zhu H Zhou Z Sharma S Liu S . Inhibition of glutamate-to-glutathione flux promotes tumor antigen presentation in colorectal cancer cells. Adv Sci (Weinh). (2025) 12:e2310308. doi: 10.1002/advs.202310308 39482885 PMC11714253

[71] Li X Wang Y Cai L Huang S . SMAD4 enhances the cytotoxic efficacy of human NK cells against colorectal cancer cells via the m(6)A reader YTHDF2. Front Immunol. (2024) 15:1440308. doi: 10.3389/fimmu.2024.1440308 39439794 PMC11494605

[72] Tan Z Tian L Luo Y Ai K Zhang X Yuan H . Preventing postsurgical colorectal cancer relapse: A hemostatic hydrogel loaded with METTL3 inhibitor for CAR-NK cell therapy. Bioact Mater. (2025) 44:236–55. doi: 10.1016/j.bioactmat.2024.10.015 39497707 PMC11532749

[73] Aashaq S Batool A Mir SA Beigh MA Andrabi KI Shah ZA . TGF-β signaling: A recap of SMAD-independent and SMAD-dependent pathways. J Cell Physiol. (2022) 237:59–85. doi: 10.1002/jcp.30529 34286853

[74] Xiao G Zheng Y Chen H Luo M Yang C Ren D . Single-cell transcriptome analysis reveals immunosuppressive landscape in overweight and obese colorectal cancer. J Transl Med. (2024) 22:134. doi: 10.1186/s12967-024-04921-5 38311726 PMC10838453

[75] She X Wu Q Rao Z Song D Huang C Feng S . SETDB1 methylates MCT1 promoting tumor progression by enhancing the lactate shuttle. Adv Sci (Weinh). (2023) 10:e2301871. doi: 10.1002/advs.202301871 37541664 PMC10558670

[76] Huang B Yu Z Cui D Du F . MAPKAP1 orchestrates macrophage polarization and lipid metabolism in fatty liver-enhanced colorectal cancer. Transl Oncol. (2024) 45:101941. doi: 10.1016/j.tranon.2024.101941 38692197 PMC11070763

[77] Sun M Yue Y Wang X Feng H Qin Y Chen M . ALKBH5-mediated upregulation of CPT1A promotes macrophage fatty acid metabolism and M2 macrophage polarization, facilitating Malignant progression of colorectal cancer. Exp Cell Res. (2024) 437:113994. doi: 10.1016/j.yexcr.2024.113994 38479704

[78] Noy R Pollard JW . Tumor-associated macrophages: from mechanisms to therapy. Immunity. (2014) 41:49–61. doi: 10.1016/j.immuni.2014.06.010 25035953 PMC4137410

[79] He S Song W Cui S Li J Jiang Y Chen X . Modulation of miR-146b by N6-methyladenosine modification remodels tumor-associated macrophages and enhances anti-PD-1 therapy in colorectal cancer. Cell Oncol (Dordr). (2023) 46:1731–46. doi: 10.1007/s13402-023-00839-0 37402945 PMC10697876

[80] Wang X Luo X Chen C Tang Y Li L Mo B . The Ap-2α/Elk-1 axis regulates Sirpα-dependent tumor phagocytosis by tumor-associated macrophages in colorectal cancer. Signal Transduct Target Ther. (2020) 5:35. doi: 10.1038/s41392-020-0124-z 32296015 PMC7156469

[81] Zheng H Yu S Zhu C Guo T Liu F Xu Y . HIF1α promotes tumor chemoresistance via recruiting GDF15-producing TAMs in colorectal cancer. Exp Cell Res. (2021) 398:112394. doi: 10.1016/j.yexcr.2020.112394 33242463

[82] Abdalsalam NMF Ibrahim A Saliu MA Liu TM Wan X Yan D . MDSC: a new potential breakthrough in CAR-T therapy for solid tumors. Cell Commun Signal. (2024) 22:612. doi: 10.1186/s12964-024-01995-y 39702149 PMC11660884

[83] Zhang Z Zheng Y Chen Y Yin Y Chen Y Chen Q . Gut fungi enhances immunosuppressive function of myeloid-derived suppressor cells by activating PKM2-dependent glycolysis to promote colorectal tumorigenesis. Exp Hematol Oncol. (2022) 11:88. doi: 10.1186/s40164-022-00334-6 36348389 PMC9644472

[84] Hayes C Donohoe CL Davern M Donlon NE . The oncogenic and clinical implications of lactate induced immunosuppression in the tumour microenvironment. Cancer Lett. (2021) 500:75–86. doi: 10.1016/j.canlet.2020.12.021 33347908

[85] Nicolini A Ferrari P . Involvement of tumor immune microenvironment metabolic reprogramming in colorectal cancer progression, immune escape, and response to immunotherapy. Front Immunol. (2024) 15:1353787. doi: 10.3389/fimmu.2024.1353787 39119332 PMC11306065

[86] Husain Z Seth P Sukhatme VP . Tumor-derived lactate and myeloid-derived suppressor cells: Linking metabolism to cancer immunology. Oncoimmunology. (2013) 2:e26383. doi: 10.4161/onci.26383 24404426 PMC3881600

[87] Chen H Pan Y Zhou Q Liang C Wong CC Zhou Y . METTL3 inhibits antitumor immunity by targeting m(6)A-BHLHE41-CXCL1/CXCR2 axis to promote colorectal cancer. Gastroenterology. (2022) 163:891–907. doi: 10.1053/j.gastro.2022.06.024 35700773

[88] Xiong J He J Zhu J Pan J Liao W Ye H . Lactylation-driven METTL3-mediated RNA m(6)A modification promotes immunosuppression of tumor-infiltrating myeloid cells. Mol Cell. (2022) 82:1660–77:e10. doi: 10.1016/j.molcel.2022.02.033 35320754

[89] Li C Jiang P Wei S Xu X Wang J . Regulatory T cells in tumor microenvironment: new mechanisms, potential therapeutic strategies and future prospects. Mol Cancer. (2020) 19:116. doi: 10.1186/s12943-020-01234-1 32680511 PMC7367382

[90] Sakaguchi S Mikami N Wing JB Tanaka A Ichiyama K Ohkura N . Regulatory T cells and human disease. Annu Rev Immunol. (2020) 38:541–66. doi: 10.1146/annurev-immunol-042718-041717 32017635

[91] Toker A Ohashi PS . Expression of costimulatory and inhibitory receptors in FoxP3(+) regulatory T cells within the tumor microenvironment: Implications for combination immunotherapy approaches. Adv Cancer Res. (2019) 144:193–261. doi: 10.1016/bs.acr.2019.05.001 31349899

[92] Angelin A Gil-de-Gomez L Dahiya S Jiao J Guo L Levine MH . Foxp3 reprograms T cell metabolism to function in low-glucose, high-lactate environments. Cell Metab. (2017) 25:1282–93:e7. doi: 10.1016/j.cmet.2016.12.018 28416194 PMC5462872

[93] Watson MJ Vignali PDA Mullett SJ Overacre-Delgoffe AE Peralta RM Grebinoski S . Metabolic support of tumour-infiltrating regulatory T cells by lactic acid. Nature. (2021) 591:645–51. doi: 10.1038/s41586-020-03045-2 33589820 PMC7990682

[94] Gu J Zhou J Chen Q Xu X Gao J Li X . Tumor metabolite lactate promotes tumorigenesis by modulating MOESIN lactylation and enhancing TGF-beta signaling in regulatory T cells. Cell Rep. (2022) 40:111122. doi: 10.1016/j.celrep.2022.111122 35858574

[95] Kumagai S Koyama S Itahashi K Tanegashima T Lin YT Togashi Y . Lactic acid promotes PD-1 expression in regulatory T cells in highly glycolytic tumor microenvironments. Cancer Cell. (2022) 40:201–18:e9. doi: 10.1016/j.ccell.2022.01.001 35090594

[96] Xue R Zhang Q Cao Q Kong R Xiang X Liu H . Liver tumour immune microenvironment subtypes and neutrophil heterogeneity. Nature. (2022) 612:141–7. doi: 10.1038/s41586-022-05400-x 36352227

[97] Qian L Ji Z Mei L Zhao J . IGF2BP2 promotes lung adenocarcinoma progression by regulating LOX1 and tumor-associated neutrophils. Immunol Res. (2024) 73:16. doi: 10.1007/s12026-024-09563-9 39688738

[98] He J Zhou M Yin J Wan J Chu J Jia J . METTL3 restrains papillary thyroid cancer progression via m(6)A/c-Rel/IL-8-mediated neutrophil infiltration. Mol Ther. (2021) 29:1821–37. doi: 10.1016/j.ymthe.2021.01.019 33484966 PMC8116572

[99] Liu Y Song R Zhao L Lu Z Li Y Zhan X . m(6)A demethylase ALKBH5 is required for antibacterial innate defense by intrinsic motivation of neutrophil migration. Signal Transduct Target Ther. (2022) 7:194. doi: 10.1038/s41392-022-01020-z 35764614 PMC9240034

[100] Wang L Liu Y Dai Y Tang X Yin T Wang C . Single-cell RNA-seq analysis reveals BHLHE40-driven pro-tumour neutrophils with hyperactivated glycolysis in pancreatic tumour microenvironment. Gut. (2023) 72:958–71. doi: 10.1136/gutjnl-2021-326070 35688610 PMC10086491

[101] Chen Y McAndrews KM Kalluri R . Clinical and therapeutic relevance of cancer-associated fibroblasts. Nat Rev Clin Oncol. (2021) 18:792–804. doi: 10.1038/s41571-021-00546-5 34489603 PMC8791784

[102] Wang J Delfarah A Gelbach PE Fong E Macklin P Mumenthaler SM . Elucidating tumor-stromal metabolic crosstalk in colorectal cancer through integration of constraint-based models and LC-MS metabolomics. Metab Eng. (2022) 69:175–87. doi: 10.1016/j.ymben.2021.11.006 34838998 PMC8818109

[103] Bertero T Oldham WM Grasset EM Bourget I Boulter E Pisano S . Tumor-stroma mechanics coordinate amino acid availability to sustain tumor growth and Malignancy. Cell Metab. (2019) 29:124–140.e10. doi: 10.1016/j.cmet.2018.09.012 30293773 PMC6432652

[104] Gong J Lin Y Zhang H Liu C Cheng Z Yang X . Reprogramming of lipid metabolism in cancer-associated fibroblasts potentiates migration of colorectal cancer cells. Cell Death Dis. (2020) 11:267. doi: 10.1038/s41419-020-2434-z 32327627 PMC7181758

[105] Strating E Verhagen MP Wensink E Dünnebach E Wijler L Aranguren I . Co-cultures of colon cancer cells and cancer-associated fibroblasts recapitulate the aggressive features of mesenchymal-like colon cancer. Front Immunol. (2023) 14:1053920. doi: 10.3389/fimmu.2023.1053920 37261365 PMC10228738

[106] Ren H Wang M Ma X An L Guo Y Ma H . METTL3 in cancer-associated fibroblasts-derived exosomes promotes the proliferation and metastasis and suppresses ferroptosis in colorectal cancer by eliciting ACSL3 m6A modification. Biol Direct. (2024) 19:68. doi: 10.1186/s13062-024-00511-z 39160584 PMC11331890

[107] Liao JN Ni WJ Wu PH Yang YD Yang Y Long W . Switching from messenger RNAs to noncoding RNAs, METTL3 is a novel colorectal cancer diagnosis and treatment target. World J Gastrointest Oncol. (2025) 17:104076. doi: 10.4251/wjgo.v17.i5.104076 40487941 PMC12142237

[108] Xie J Huang Z Jiang P Wu R Jiang H Luo C . Elevated N6-methyladenosine RNA levels in peripheral blood immune cells: A novel predictive biomarker and therapeutic target for colorectal cancer. Front Immunol. (2021) 12:760747. doi: 10.3389/fimmu.2021.760747 34659267 PMC8515146

[109] Li T Hu PS Zuo Z Lin JF Li X Wu QN . METTL3 facilitates tumor progression via an m(6)A-IGF2BP2-dependent mechanism in colorectal carcinoma. Mol Cancer. (2019) 18:112. doi: 10.1186/s12943-019-1038-7 31230592 PMC6589893

[110] Chen X Xu M Xu X Zeng K Liu X Pan B . METTL14-mediated N6-methyladenosine modification of SOX4 mRNA inhibits tumor metastasis in colorectal cancer. Mol Cancer. (2020) 19:106. doi: 10.1186/s12943-020-01220-7 32552762 PMC7298962

[111] Yang X Zhang S He C Xue P Zhang L He Z . METTL14 suppresses proliferation and metastasis of colorectal cancer by down-regulating oncogenic long non-coding RNA XIST. Mol Cancer. (2020) 19:46. doi: 10.1186/s12943-020-1146-4 32111213 PMC7047419

[112] Tan YT Li T Wang RB Liu ZK Ma MY Huang RZ . WTAP weakens oxaliplatin chemosensitivity of colorectal cancer by preventing PANoptosis. Cancer Lett. (2024) 604:217254. doi: 10.1016/j.canlet.2024.217254 39270768

[113] Ruan DY Li T Wang YN Meng Q Li Y Yu K . FTO downregulation mediated by hypoxia facilitates colorectal cancer metastasis. Oncogene. (2021) 40:5168–81. doi: 10.1038/s41388-021-01916-0 34218271 PMC8376648

[114] Zhang Z Wang L Zhao L Wang Q Yang C Zhang M . N6-methyladenosine demethylase ALKBH5 suppresses colorectal cancer progression potentially by decreasing PHF20 mRNA methylation. Clin Transl Med. (2022) 12:e940. doi: 10.1002/ctm2.940 35979628 PMC9386323

[115] Li W Gao Y Jin X Wang H Lan T Wei M . Comprehensive analysis of N6-methylandenosine regulators and m6A-related RNAs as prognosis factors in colorectal cancer. Mol Ther Nucleic Acids. (2022) 27:598–610. doi: 10.1016/j.omtn.2021.12.007 35070494 PMC8753275

[116] Kandimalla R Gao F Li Y Huang H Ke J Deng X . RNAMethyPro: a biologically conserved signature of N6-methyladenosine regulators for predicting survival at pan-cancer level. NPJ Precis Oncol. (2019) 3:13. doi: 10.1038/s41698-019-0085-2 31069256 PMC6494854

[117] Yue Q Zhang Y Wang F Cao F Bai J Duan X . Characterization of m6A methylation modification patterns in colorectal cancer determines prognosis and tumor microenvironment infiltration. J Immunol Res. (2022) 2022:8766735. doi: 10.1155/2022/8766735 35692505 PMC9177296

[118] Li J Liang L Yang Y Li X Ma Y . N(6)-methyladenosine as a biological and clinical determinant in colorectal cancer: progression and future direction. Theranostics. (2021) 11:2581–93. doi: 10.7150/thno.52366 33456561 PMC7806471

[119] Sun Y Zhang X Hang D Lau HC Du J Liu C . Integrative plasma and fecal metabolomics identify functional metabolites in adenoma-colorectal cancer progression and as early diagnostic biomarkers. Cancer Cell. (2024) 42:1386–1400.e8. doi: 10.1016/j.ccell.2024.07.005 39137727

[120] Răchieriu C Eniu DT Moiş E Graur F Socaciu C Socaciu MA . Lipidomic signatures for colorectal cancer diagnosis and progression using UPLC-QTOF-ESI(+)MS. Biomolecules. (2021) 11:417. doi: 10.3390/biom11030417 33799830 PMC8035671

[121] Dai Z Li T Lai K Wang X Zhou P Hu K . Serum metabolic characteristics associated with the deterioration of colorectal adenomas. Sci Rep. (2025) 15:6845. doi: 10.1038/s41598-025-91444-8 40000732 PMC11861597

[122] Uchiyama K Yagi N Mizushima K Higashimura Y Hirai Y Okayama T . Serum metabolomics analysis for early detection of colorectal cancer. J Gastroenterol. (2017) 52:677–94. doi: 10.1007/s00535-016-1261-6 27650200

[123] Geijsen A van Roekel EH van Duijnhoven FJB Achaintre D Bachleitner-Hofmann T Baierl A . Plasma metabolites associated with colorectal cancer stage: Findings from an international consortium. Int J Cancer. (2020) 146:3256–66. doi: 10.1002/ijc.32666 31495913 PMC7216900

[124] Nishiumi S Kobayashi T Ikeda A Yoshie T Kibi M Izumi Y . A novel serum metabolomics-based diagnostic approach for colorectal cancer. PloS One. (2012) 7:e40459. doi: 10.1371/journal.pone.0040459 22792336 PMC3394708

[125] Di Donato S Vignoli A Biagioni C Malorni L Mori E Tenori L . A serum metabolomics classifier derived from elderly patients with metastatic colorectal cancer predicts relapse in the adjuvant setting. Cancers (Basel). (2021) 13:2762. doi: 10.3390/cancers13112762 34199435 PMC8199587

[126] Zha H Cai Y Yin Y Wang Z Li K Zhu ZJ . SWATHtoMRM: Development of high-coverage targeted metabolomics method using SWATH technology for biomarker discovery. Anal Chem. (2018) 90:4062–70. doi: 10.1021/acs.analchem.7b05318 29485856

[127] Jia H Shen X Guan Y Xu M Tu J Mo M . Predicting the pathological response to neoadjuvant chemoradiation using untargeted metabolomics in locally advanced rectal cancer. Radiother Oncol. (2018) 128:548–56. doi: 10.1016/j.radonc.2018.06.022 30041962

[128] Xie Y Jin Y Liu Z Li J Tao Q Wu Y . Identification of diagnostic biomarkers for colorectal polyps based on noninvasive urinary metabolite screening and construction of a nomogram. Cancer Med. (2025) 14:e70762. doi: 10.1002/cam4.70762 40200572 PMC11978731

[129] Ning W Qiao N Zhang X Pei D Wang W . Metabolic profiling analysis for clinical urine of colorectal cancer. Asia Pac J Clin Oncol. (2021) 17:403–13. doi: 10.1111/ajco.13591 34164923

[130] Nannini G Meoni G Tenori L Ringressi MN Taddei A Niccolai E . Fecal metabolomic profiles: A comparative study of patients with colorectal cancer vs adenomatous polyps. World J Gastroenterol. (2021) 27:6430–41. doi: 10.3748/wjg.v27.i38.6430 34720532 PMC8517777

[131] Song EM Byeon JS Lee SM Yoo HJ Kim SJ Lee SH . Fecal fatty acid profiling as a potential new screening biomarker in patients with colorectal cancer. Dig Dis Sci. (2018) 63:1229–36. doi: 10.1007/s10620-018-4982-y 29516324

[132] Clos-Garcia M Garcia K Alonso C Iruarrizaga-Lejarreta M D'Amato M Crespo A . Integrative analysis of fecal metagenomics and metabolomics in colorectal cancer. Cancers (Basel). (2020) 12. doi: 10.3390/cancers12051142 32370168 PMC7281174

[133] Cai R Ke L Zhao Y Zhao J Zhang H Zheng P . NMR-based metabolomics combined with metabolic pathway analysis reveals metabolic heterogeneity of colorectal cancer tissue at different anatomical locations and stages. Int J Cancer. (2025) 156:1644–55. doi: 10.1002/ijc.35273 39629979 PMC11826128

[134] Redalen KR Sitter B Bathen TF Grøholt KK Hole KH Dueland S . High tumor glycine concentration is an adverse prognostic factor in locally advanced rectal cancer. Radiother Oncol. (2016) 118:393–8. doi: 10.1016/j.radonc.2015.11.031 26705680

[135] Tian Y Xu T Huang J Zhang L Xu S Xiong B . Tissue metabonomic phenotyping for diagnosis and prognosis of human colorectal cancer. Sci Rep. (2016) 6:20790. doi: 10.1038/srep20790 26876567 PMC4753490

[136] Dalal N Jalandra R Bayal N Yadav AK Harshulika Sharma M . Gut microbiota-derived metabolites in CRC progression and causation. J Cancer Res Clin Oncol. (2021) 147:3141–55. doi: 10.1007/s00432-021-03729-w 34273006 PMC11801839

[137] Zhang R Li M Tan H Liu J Wang L Dai W . A new gut pathogenic bacteria and its metabolites promote colorectal cancer development and act as non-invasive early diagnostic biomarkers. Gut Microbes. (2025) 17:2555446. doi: 10.1080/19490976.2025.2555446 40911847 PMC12416176

[138] Hou Y Wu H Zhang Z Wang J Chen Q Lian C . Bacteroides intestinalis mediates the sensitivity to irinotecan toxicity via tryptophan catabolites. Gut. (2025) 75:1322–37. doi: 10.1136/gutjnl-2024-334699 40903035

[139] Kim JS Cho S Jeong MY Rivera-Piza A Kim Y Wu C . β-Ionone suppresses colorectal tumorigenesis by activating OR51E2, a potential tumor suppressor. Phytomedicine. (2025) 140:156599. doi: 10.1016/j.phymed.2025.156599 40088737

[140] Deng A Fan R Hai Y Zhuang J Zhang B Lu X . A STING agonist prodrug reprograms tumor-associated macrophage to boost colorectal cancer immunotherapy. Theranostics. (2025) 15:277–99. doi: 10.7150/thno.101001 39744236 PMC11667223

[141] Ji L Shen W Zhang F Qian J Jiang J Weng L . Worenine reverses the Warburg effect and inhibits colon cancer cell growth by negatively regulating HIF-1α. Cell Mol Biol Lett. (2021) 26:19. doi: 10.1186/s11658-021-00263-y 34006215 PMC8130299

[142] Mao L Chen Q Gong K Xu X Xie Y Zhang W . Berberine decelerates glucose metabolism via suppression of mTOR-dependent HIF-1α protein synthesis in colon cancer cells. Oncol Rep. (2018) 39:2436–42. doi: 10.3892/or.2018.6318 29565467

[143] Hong X Zhong L Xie Y Zheng K Pang J Li Y . Matrine reverses the Warburg effect and suppresses colon cancer cell growth via negatively regulating HIF-1α. Front Pharmacol. (2019) 10:1437. doi: 10.3389/fphar.2019.01437 31849679 PMC6892950

[144] Kim HS Wannatung T Lee S Yang WK Chung SH Lim JS . Quercetin enhances hypoxia-mediated apoptosis via direct inhibition of AMPK activity in HCT116 colon cancer. Apoptosis. (2012) 17:938–49. doi: 10.1007/s10495-012-0719-0 22684842

[145] Xu Y Han S Lei K Chang X Wang K Li Z . Anti-Warburg effect of rosmarinic acid via miR-155 in colorectal carcinoma cells. Eur J Cancer Prev. (2016) 25:481–9. doi: 10.1097/cej.0000000000000205 26340059

[146] Ni T He Z Dai Y Yao J Guo Q Wei L . Oroxylin A suppresses the development and growth of colorectal cancer through reprogram of HIF1α-modulated fatty acid metabolism. Cell Death Dis. (2017) 8:e2865. doi: 10.1038/cddis.2017.261 28594405 PMC5520917

[147] Maione F Oliaro-Bosso S Meda C Di Nicolantonio F Bussolino F Balliano G . The cholesterol biosynthesis enzyme oxidosqualene cyclase is a new target to impair tumour angiogenesis and metastasis dissemination. Sci Rep. (2015) 5:9054. doi: 10.1038/srep09054 25761781 PMC4357009

[148] Vijayan Y James S Viswanathan A Aparna JS Bindu A Namitha NN . Targeting acid ceramidase enhances antitumor immune response in colorectal cancer. J Adv Res. (2024) 65:73–87. doi: 10.1016/j.jare.2023.12.013 38142035 PMC11518951

[149] Tsenkova M Brauer M Pozdeev VI Kasakin M Busi SB Schmoetten M . Ketogenic diet suppresses colorectal cancer through the gut microbiome long chain fatty acid stearate. Nat Commun. (2025) 16:1792. doi: 10.1038/s41467-025-56678-0 39979287 PMC11842570

[150] Mi X Duan Y Sun J Tai Q Yao H Meng L . The ketogenic diet modulates tumor-associated neutrophil polarization via the AMOT-YAP/TAZ axis to inhibit colorectal cancer progression. Pharmacol Res. (2024) 210:107494. doi: 10.1016/j.phrs.2024.107494 39510146

[151] Spada M Piras C Leoni VP Casula M Simbula G Noto A . Glutaminase inhibitor CB-839 causes metabolic adjustments in colorectal cancer cells. Sci Rep. (2025) 15:37028. doi: 10.1038/s41598-025-20528-2 41131154 PMC12550074

[152] Chen Z Xu J Fang K Jiang H Leng Z Wu H . FOXC1-mediated serine metabolism reprogramming enhances colorectal cancer growth and 5-FU resistance under serine restriction. Cell Commun Signal. (2025) 23:13. doi: 10.1186/s12964-024-02016-8 39773485 PMC11708197

[153] Tong H Jiang Z Song L Tan K Yin X He C . Dual impacts of serine/glycine-free diet in enhancing antitumor immunity and promoting evasion via PD-L1 lactylation. Cell Metab. (2024) 36:2493–2510.e9. doi: 10.1016/j.cmet.2024.10.019 39577415

[154] Wang QL Chen Z Lu X Lin H Feng H Weng N . Methionine metabolism dictates PCSK9 expression and antitumor potency of PD-1 blockade in MSS colorectal cancer. Adv Sci (Weinh). (2025) 12:e2501623. doi: 10.1002/advs.202501623 40125618 PMC12097065

[155] Ren YM Zhuang ZY Xie YH Yang PJ Xia TX Xie YL . BCAA-producing Clostridium symbiosum promotes colorectal tumorigenesis through the modulation of host cholesterol metabolism. Cell Host Microbe. (2024) 32:1519–1535.e7. doi: 10.1016/j.chom.2024.07.012 39106870

[156] Xu H Wang Y Liu G Zhu Z Shahbazi MA Reis RL . Nano-Armed Limosilactobacillus reuteri for enhanced photo-immunotherapy and microbiota tryptophan metabolism against colorectal cancer. Adv Sci (Weinh). (2025) 12:e2410011. doi: 10.1002/advs.202410011 39739630 PMC11831460

[157] Zhou Y Tao Q Luo C Chen J Chen G Sun J . Epacadostat overcomes cetuximab resistance in colorectal cancer by targeting IDO-mediated tryptophan metabolism. Cancer Sci. (2025) 116:1715–29. doi: 10.1111/cas.70057 40103010 PMC12127106

[158] Yang Z Quan Y Chen Y Huang Y Huang R Yu W . Knockdown of RNA N6-methyladenosine methyltransferase METTL3 represses Warburg effect in colorectal cancer via regulating HIF-1alpha. Signal Transduct Target Ther. (2021) 6:89. doi: 10.1038/s41392-021-00473-y 33637677 PMC7910535

[159] Wang J Deng J Sun B Qiao G Yang J Gao Z . Gambogic acid induces ferroptosis and suppresses colorectal cancer progression by modulating the m6A modification of p62. Cell Signal. (2025) 135:112024. doi: 10.1016/j.cellsig.2025.112024 40721064

[160] Hu A Wang H Xu Q Pan Y Jiang Z Li S . A novel CPT1A covalent inhibitor modulates fatty acid oxidation and CPT1A-VDAC1 axis with therapeutic potential for colorectal cancer. Redox Biol. (2023) 68:102959. doi: 10.1016/j.redox.2023.102959 37977042 PMC10692921

[161] Tao ZH Han JX Xu J Zhao E Wang M Wang Z . Screening of patient-derived organoids identifies mitophagy as a cell-intrinsic vulnerability in colorectal cancer during statin treatment. Cell Rep Med. (2025) 6:102039. doi: 10.1016/j.xcrm.2025.102039 40154491 PMC12047522

[162] Xiong L Yang X Liu H Wu X Cai T Yuan M . Glutamic-pyruvic transaminase 1 deficiency-mediated metabolic reprogramming facilitates colorectal adenoma-carcinoma progression. Sci Transl Med. (2025) 17:eadp9805. doi: 10.1126/scitranslmed.adp9805 39742507

